# The *I* gene defines a dynamic *NLR* cluster conferring broad potyvirus resistance in common bean

**DOI:** 10.1038/s41467-026-73550-x

**Published:** 2026-05-30

**Authors:** Juan C. Alvarez-Díaz, Alvaro Soler-Garzón, Gianluca Teano, Marion Verdenaud, Alexandre Roudaut, Christophe Klopp, Maria-Victoria Préjean, Stéphanie Pflieger, Timothy G. Porch, Cécile Desbiez, David Latrasse, Moussa Benhamed, Etienne Delannoy, Andrea Pedrosa-Harand, Ariane Gratias, Phillip N. Miklas, Valérie Geffroy

**Affiliations:** 1 Université Paris-Saclay, CNRS, INRAE, Université Evry, Institute of Plant Sciences Paris-Saclay (IPS2), Gif sur Yvette, France; 2 Université Paris Cité, CNRS, INRAE, Institute of Plant Sciences Paris-Saclay (IPS2), Orsay, France; 3 Irrigated Agriculture Research and Extension Center, Washington State University, Prosser, WA USA; 4 Université de Toulouse, GenoToul Bioinformatic Facility, Sigenae, BioinfOmics, UR875 MIAT, INRAE, Castanet-Tolosan, France; 5 Tropical Agricultural Research Station, United States Department of Agriculture - Agricultural Research Service (USDA-ARS), Mayagüez, Puerto Rico, PR USA; 6 INRAE, Pathologie Végétale, Montfavet, France; 7 Laboratory of Plant Cytogenetics and Evolution, Department of Botany, Biosciences Centre, Federal University of Pernambuco, Recife, Brazil; 8 Grain Legume Genetics and Physiology Research Unit, United States Department of Agriculture - Agricultural Research Service (USDA-ARS), Prosser, WA USA

**Keywords:** Agricultural genetics, Biotic, Genetic variation, Plant breeding

## Abstract

Common bean (***Phaseolus vulgaris***) is a major grain legume for human consumption, but its production is severely constrained by viral diseases, especially those caused by bean common mosaic virus and bean common mosaic necrosis virus. The function of the dominant ***I*** gene conferring broad-spectrum resistance to potyviruses has been known for nearly a century, yet its molecular identity remains unresolved. Here, we combine chromosome-scale genome assemblies and two loss-of-function mutants to clone the ***I*** gene and show that it encodes a Toll/interleukin-1 receptor-like nucleotide-binding leucine-rich-repeat (NLR) (TNL) protein. ***I*** resides in a dynamic TNL-rich resistance cluster exhibiting dramatic differences in TNL copy number among genotypes. In one natural mutant, resistance is lost through the recent insertion of a non-autonomous Retand retrotransposon, providing a rare example of transposon-mediated *R* gene inactivation during seed propagation. More broadly, we show that ***Phaseolus*** genomes are enriched in Retand elements, representing a distinct legume genome evolution trajectory comparing to pea and faba bean. These findings resolve a long-standing question in common bean genetics and open avenues for crop improvement.

## Abstract (web summary)

Potyviruses seriously affect common bean production worldwide. Here, the authors combine genome assembly and characterization of loss-of-function mutants to reveal a dynamic *NLR* cluster conferring durable and broad-spectrum potyvirus resistance in common bean.

## Introduction

Plants are constantly challenged by diverse pathogenic organisms, including viruses, bacteria, fungi, oomycetes, herbivores and parasitic plants. Against these threats, they have evolved a sophisticated immune system centered around intracellular immune receptors known as nucleotide-binding leucine-rich repeat (NLR) proteins^1^. After direct or indirect recognition of pathogen effectors, NLRs activate immune signaling pathways that often culminate in a localized programmed cell death response known as the hypersensitive response (HR)^1^. Based on their N-terminal domain, NLRs are classified into three main subfamilies: Toll/interleukin-1 receptor NLRs (TNLs), coiled-coil NLRs (CNLs), and RPW8-like NLRs (RNLs)^1^. These genes are often organized into clusters within plant genomes, a feature believed to promote unequal recombination, gene conversion and segmental duplications, fostering evolutionary innovation in immune specificity^2–5^. Chemical control of viral diseases in plants remains largely ineffective, positioning genetic resistance, mediated by resistance (*R*) genes, as an effective and environment-friendly strategy. Therefore, the identification and cloning of antiviral *NLRs* are essential for ensuring sustainable agricultural practices.

Common bean (*Phaseolus vulgaris* L.) is a key staple crop in many low- and middle-income countries, especially in Latin America, its center of domestication^6^, and Eastern Africa, where it contributes from 10% up to 35% of daily protein intake among vulnerable populations^7^. In high-income countries, it is also gaining in importance as a nutrient dense fiber and protein source, offering health benefits, such as promoting gut health, mitigating obesity, and reducing risk of cardiovascular disease^8,9^.

A major constraint to common bean production is bean common mosaic disease, caused by two closely related potyviruses: bean common mosaic virus (BCMV, *Potyvirus phaseovulgaris*) and bean common mosaic necrosis virus (BCMNV, *P. phaseoli*). Since their discovery in 1918^10^, these seed-transmissible viruses have spread throughout common bean-production regions worldwide, becoming one of the most destructive common bean diseases. Genetic resistance, particularly through the deployment of the *I* gene, has provided an effective and sustainable strategy for disease control. Initially discovered nearly one century ago by Mr. Corbett^11^ and later characterized as a dominant gene^12^, *I* remains the only known dominant *R* gene conferring resistance to these potyviruses in *P. vulgaris* genetic resources^13^. Despite decades of widespread use, no strains have emerged that can overcome *I*-mediated resistance, as to date mosaic symptoms on *I* bearing genotypes have never been reported, underscoring its exceptional durability.

The resistance phenotype conferred by the *I* gene is complex and varies according to the viral strain and the temperature. At temperatures below 30 °C, it provides high-level or complete (extreme) resistance to BCMV strains, which has led to its use throughout the world in breeding programs. However, BCMV strains can trigger temperature-dependent systemic necrosis in *I*-carrying genotypes when the temperature exceeds 30 °C. Additionally, strains of BCMNV induce systemic necrosis at all temperatures, complicating the deployment of *I*-based resistance in breeding programs.

Genetic mapping has placed the *I* gene at the distal end of chromosome 2, within a complex, multigenic resistance cluster that provides protection against a broad spectrum of pathogens, including nine potyviruses [ZYMV (zucchini yellow mosaic virus, *P. cucurbitaflavitesselati*), PWV (passionfruit woodiness virus, *P. passiflorae*), WMV (watermelon mosaic virus, *P. citrulli*), CABMV (cowpea aphid-borne mosaic virus, *P. vignae*), ThPV (Thailand passiflora virus, undefined species –possibly East Asian passiflora virus, EAPV, *P. orionspassiflorae*), SMV (soybean mosaic virus, *P. glycitessellati*), BYMV (bean yellow mosaic virus, *P. phaseoluteum*), ClYVV (clover yellow vein virus, *P. trifolii*)], at least two comoviruses [BPMV (bean pod mottle virus, *Comovirus siliquae*) and South American legume-infecting comovirus isolates initially collectively designed as “bean severe mosaic virus”], as well as against the bacterium *Pseudomonas syringae* pv. *phaseolicola* and the fungus *Colletotrichum lindemuthianum*^14–19^. Molecular studies have shown that the *I* locus co-localizes with a cluster of TNL genes^20–23^, but it has remained unclear whether resistance is conferred by a single broad-spectrum gene or by multiple, tightly linked genes with distinct specificities.

Despite the significant agronomic value of this antiviral *R* gene, the molecular characterization of the *I* gene cluster has been challenging due to its inherent complexity. Indeed, the presence of repeated sequences and the suppression of recombination in this region has hindered the application of traditional positional cloning strategies^22,23^. Recent breakthroughs in long-read sequencing technologies have revolutionized our ability to resolve complex genomic regions in plants, offering opportunities to decipher the molecular basis of the *I* gene^24^.

In this study, we present high-quality, chromosome-level assemblies for two widely used *P. vulgaris* genotypes, BAT93 and JaloEEP558, of Meso-American and Andean origin, respectively. These assemblies enable us to resolve the complex TNL-rich *I* gene cluster and uncover dramatic copy number variation between genotypes. Using two independent BAT93 mutants, we identify the *I* gene as a TNL conferring broad-spectrum resistance to four potyviruses. Unexpectedly, we discover that a recent insertion of a non-autonomous Retand LTR retrotransposon disrupts *I* function in a natural BAT93 mutant, revealing a case of TE-mediated resistance loss. Our findings provide the functional characterization of the *I* gene and uncover a dynamic resistance cluster shaped by structural variation.

## Results

### Improved assembly and annotation of two common bean genotypes

**Fig. 1:**
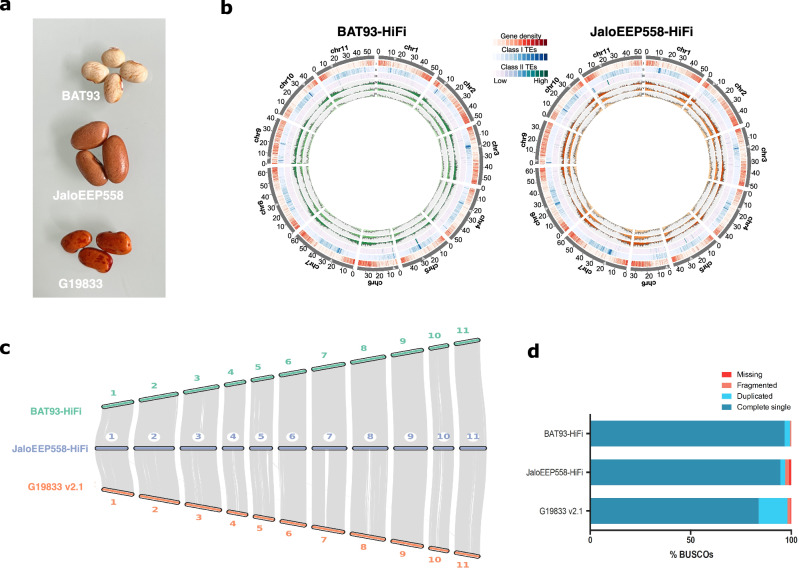
Genome assembly of genotypes BAT93 and JaloEEP558 and synteny analysis in common bean. **a** *P. vulgaris* seeds of the sequenced genotypes. The scale bar represents 1 cm. **b** Circos representation of assembled genomes of BAT93-HiFi and JaloEEP558-HiFi. The different tracks represent I) Gene density (genes per 50 kb bins), II) Class I transposons density (TEs per 50 kb bins), III) Class II transposons density (TEs per 50 kb bins), (IV–VI) Gene expression in leaf (10 days old), stem (35 days old) and root (14 days old) tissues respectively (counts per 100 Kb bins), arrowheads in the outermost circle indicate approximate centromere positions (Supplementary Data 4). **c** Syntenic relationship between the assembled genomes of BAT93-HiFi, JaloEEP558-HiFi and the reference genome of G19833 v2.1. **d** BUSCO score of the gene annotation of the sequenced and reference common bean genomes. Source data are provided as Source Data file.

A combination of PacBio Sequel II sequencing and in situ Hi-C scaffolding were used to generate a chromosome-level assembly of two cultivated genotypes of *P. vulgaris*, BAT93 and JaloEEP558 (Fig. 1a), referred to as BAT93-HiFi and JaloEEP558-HiFi, respectively. Contig-level genome assemblies of BAT93 and JaloEEP558^25^ were assembled into chromosome-scale pseudomolecules, resulting in 11 chromosomes per genotype (Fig. 1b). The scaffold N50 sizes reached 52.3 Mb for BAT93-HiFi and 49.8 Mb for JaloEEP558-HiFi, with final assembly lengths of 569.4 Mb and 542.4 Mb, respectively (Table 1). For consistency with published common bean genome sequences, chromosome names to the pseudomolecules were assigned based on syntenic relationship with the G19833 v2.1 genome assembly^20^ (Fig. 1c).

**Table 1:** Genome assembly statistics

	Genotype		
*Assembly feature*	BAT93-HiFi	JaloEEP558-HiFi	G19833 v2.1
Total genome assembly size (Mb)	637.8	606.5	537.2
Gap content (%)	0.01	0.02	1.05
Repeat sequences (%)	50.9	49.1	48.99
Number of contigs	1441	1422	1044
Contig N50 Mb (L50 number)	8.6 (26)	13.6 (16)	1.89 (73)
Mean contig size (kb)	395.2	426.5	509.2
Median contig size (kb)	34.1	41.3	96.7
Longest contig (Mb)	36.5	45.4	12.35
Number of scaffolds	1418	1315	478
Scaffolds N50 Mb (L50 number)	52.3 (6)	49.8 (6)	49.67
Mean scaffold size (kb)	442	461	1123.9
Median scaffold size (Mb)	35.3	40.4	0.015
Longest scaffold (Mb)	63.8	62.5	63.05
Assembly size in Chromosomes (Mb)	569.4	542.4	513.97

Improved assemblies of BAT93-HiFi and JaloEEP558-HiFi were annotated using the Eugene pipeline. A similar number of protein-coding genes were predicted in both genotypes, with 28,522 genes in BAT93 and 28,129 in JaloEEP558. Annotation quality was assessed by searching for 1614 highly conserved, plant-specific single-copy orthologs from the embryophyta_odb10 database using BUSCO^26^, yielding a completeness score of 96.7% and 97.0% for BAT93-HiFi and JaloEEP558-HiFi, respectively (Fig. 1d). The genome-wide distribution of genomic and epigenomic features of the two HiFi assemblies were visualized using Circos plots (Fig. 1b). Gene regions are distributed along the chromosomes and are enriched towards distal regions, co-localizing with RNA-seq expression data from leaf, stem, and root tissues (Fig. 1b). Transposable element (TE) annotation revealed a concentration of Class I TE around the (peri)centromeric regions and proximal regions. To assess chromosome-scale collinearity, genome-wide synteny analysis was performed by comparing the two HiFi assemblies to *P. vulgaris* reference genome (G19883) and other assemblies (Supplementary Fig. 1). Overall, strong collinearity was observed between G19833 v2.1^20^, BAT93-HiFi, and JaloEEP558-HiFi (Fig. 1c, Supplementary Fig. 1a and d). BAT93-HiFi and JaloEEP558-HiFi present a high degree of collinearity (Fig. 1c, Supplementary Fig. 2), while BAT93-HiFi showed very low collinearity with the previous BAT93 assembly (phasIbeam10.0)^27^, highlighting the power of long-read sequencing technology (Supplementary Fig. 1b).

Between G19833 v2.1, BAT93-HiFi, and JaloEEP558-HiFi, most chromosomes displayed only minor rearrangements (~5 Mb) concentrated in centromeric and (peri)centromeric regions (Supplementary Fig. 1). Despite their distinct geographic origin, the three genome assemblies showed high overall similarity, with most structural differences restricted to centromeric regions. Notably, BAT93-HiFi was more similar to JaloEEP558-HiFi than to its previously available assembly (Supplementary Fig. 1c)^27^.

### Comparison of *I* cluster evolution across six *P. vulgaris* genomes

**Fig. 2:**
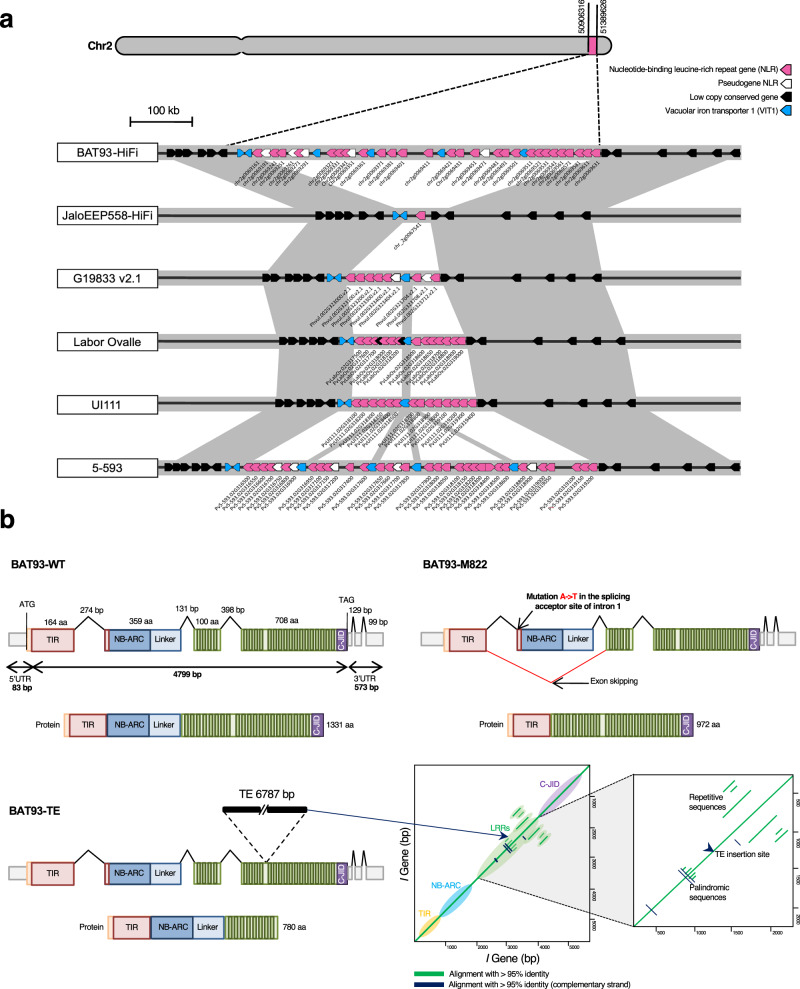
Identification and characterization of the *I* gene and its mutant alleles. **a** Physical location of the *I* cluster in BAT93-HiFi and other bean genome assemblies (JaloEEP558-HiFi, G19833 v2.1, Labor Ovalle, UI111, 5-593). The 32 annotated and manually corrected *NLRs* are indicated as full length genes (pink) and pseudogenes (white). The *I* gene is indicated by a star (*). All the *NLRs* from the *I* cluster are classified as *TNLs*. **b** *I* gene structure and predicted protein for BAT93-WT, BAT93-M822 and BAT93-TE. Dot plot of sequence comparison showing the TE insertion region within the *I* gene in BAT93-TE. The different domain regions are highlighted in colors: TIR domain (yellow), NB-ARC domain (light blue), LRR domain (light green) and the C-JID domain (purple). Source data are provided as Source Data file.

Having generated near-complete assemblies of two cultivated common bean genotypes of contrasting origin, BAT93 (Mesoamerican) and JaloEEP558 (Andean), we performed a comprehensive analysis of *NLR* clusters. Genome-wide, *NLR* clusters were located at conserved positions in both genotypes BAT93 and JaloEEP558 but showed important copy number variation (CNV). The most dramatic example is the *I* cluster region at the distal end of chromosome 2, spanning more than 700 kb and harboring 32 TNLs in BAT93-HiFi but less than 100 kb and only a single TNL in JaloEEP558-HiFi (Fig. 2a).

Taking advantage of three additional *P. vulgaris* genome assemblies, we extended our microsynteny analysis of the *I* Cluster to UI111, Labor Ovalle and 5-593 (https://phytozome-next.jgi.doe.gov), as well as to the reference genotype G19833 v2.1. Extensive TNL CNV was observed between genotypes, ranging from the single TNL in JaloEEP558, 9 in G19833, 13 in Labor Ovalle, 14 in UI111, 32 in BAT93 to 34 TNLs in 5-593 (Fig. 2a). Overall, the genotypes bearing the *I* gene (BAT93 and 5-593) present a high number of TNLs and are highly similar (Supplementary Fig. 3), while those lacking *I* gene (G19833, JaloEEP558, Labor Ovalle, UI111) are more diverse and possess fewer TNLs (Fig. 2a). For all genotypes, the TNL genes were organized in tandem arrays suggesting that unequal crossing-over contributed to their expansion. Four copies of a vacuolar iron transporter 1 encoding gene were found in 5-593 and BAT93, suggesting that segmental duplications, in addition to unequal crossing-over, may have contributed to this cluster amplification.

### Identification of the *I* gene using two independent *P. vulgaris* BAT93 mutants

**Fig. 3:**
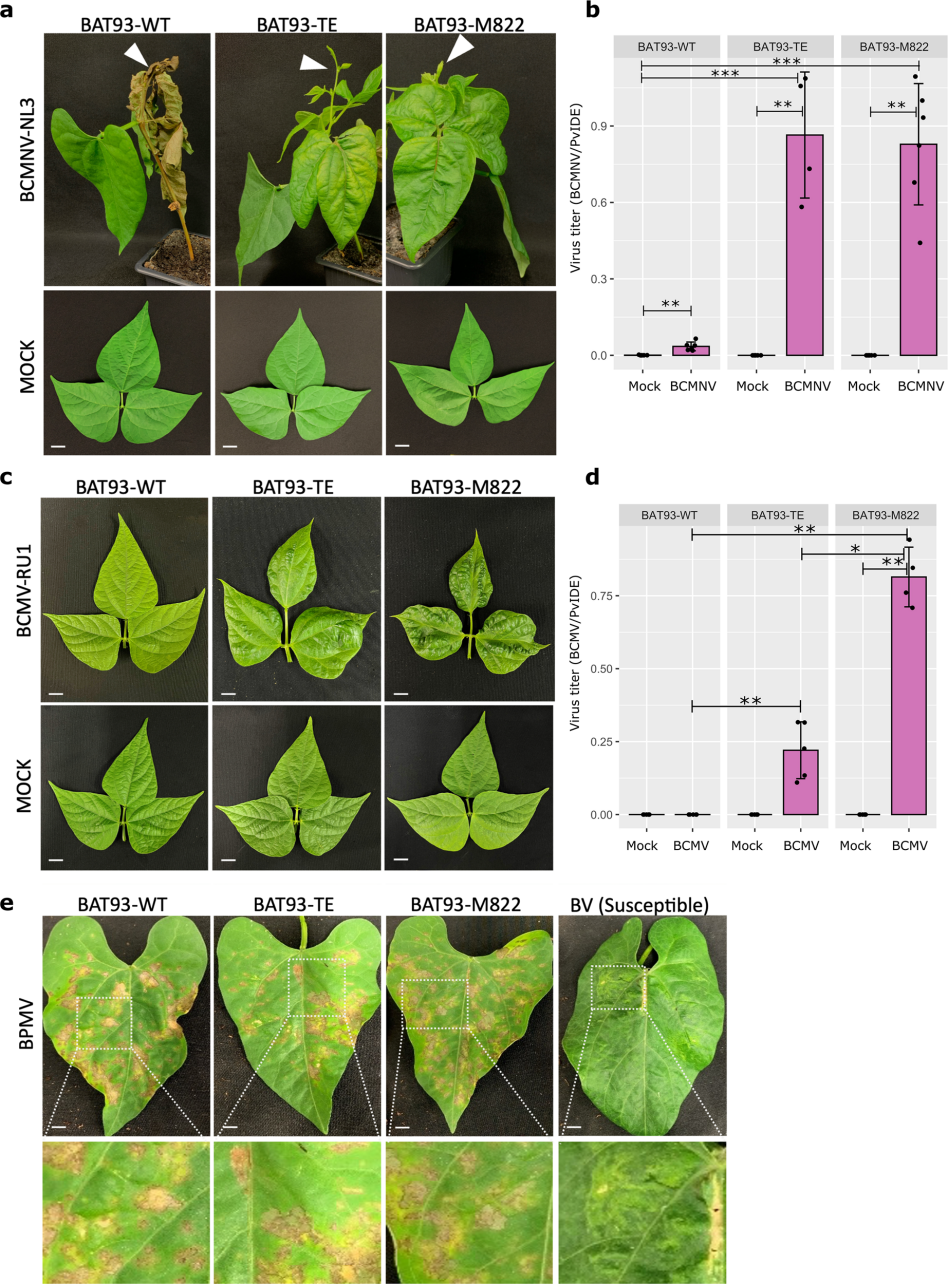
Phenotypes of infection with BCMV, BCMNV and BPMV in BAT93-WT, BAT93-TE and BAT93-M822. **a** Phenotypes on systemic leaves of BAT93-WT, and mutants BAT93-TE and BAT93-M822 at 11 days post-inoculation with BCMNV (strain NL3). For BAT93-WT systemic hypersensitive response on the top leaves corresponding to top necrosis are indicated by a white arrow. For BAT93-TE and BAT93-M822 mosaic and crinkling symptoms on the trifoliate systemic leaves and no top necrosis are indicated by white arrows. **b** RT-qPCR results of viral titer in systemic leaves (*n* = 6) from BAT93-WT and mutants at 11 days post inoculation with BCMNV (strain NL3). Black dots represent individual data points. **c** Observed phenotypes on systemic leaves from BAT93-WT and mutants at 21 days post-inoculation with BCMV (strain RU1): the systemic leaves of both mutants present typical symptoms of mosaic, including crinkling and leaf deformation, whereas no symptoms can be observed on wild-type plants (extreme resistance). **d** RT-qPCR results of viral titer in systemic leaves (*n* = 6) from BAT93-WT and mutants at 21 days post inoculation with BCMV (strain RU1). Black dots represent individual data points. **e** Observed phenotype on inoculated leaves in BAT93-WT and mutants, presenting local necrotic lesions (HR lesions) compared to Black Valentine (BV) as susceptible control with mosaic symptoms, at 7 days post inoculation with BPMV (strain IA-Di1). Scale bars = 1 cm. For b and d, data represent mean ratios ± SD of 6 biological replicates. Comparison between treatments were performed using a two-sided non-parametric Wilcoxon-Mann-Whitney U-test. *p-*values are: **b** WT vs. TE (*p* = 0.0022); WT vs. M822 (*p* = 0.0022); WT Mock vs. BCMNV (*p* = 0.0087). **d** WT vs. M822 (*p* = 0.0022); TE vs. M822 (*p* = 0.026). Asterisks indicate the level of significance: * *p* < 0.05, ** *p* < 0.01 and *** *p* < 0.001. Source data are provided as Source Data file.

To identify *I* among the candidate genes in the *I* cluster, we used an EMS mutagenized BAT93 population^28^. Screening of 1657 M3 lines with the NL3 strain of BCMNV (BCMNV-NL3) led to the identification of one mutant line, referred to as BAT93-M822, exhibiting a mutant phenotype characterized by systemic mosaic symptoms on uninoculated leaves (Fig. 3a; Supplementary Fig. 4). No systemic necrosis, also called stem apical necrosis, typical of the *I*-mediated response after infection with BCMNV was observed, suggesting that the *I* gene is not functional in BAT93-M822. The mutant phenotype of BAT93-M822 was confirmed in subsequent independent inoculations with BCMNV-NL3 (Fig. 3a).

To identify the causal mutation, BAT93-M822 was sequenced using DNA nanoballs platform (generating 75 million 150 bp paired-end reads). About 78.6% of the reads were aligned to BAT93-HiFi assembly revealing 41,310 variants in M822 across the 11 chromosomes and unanchored scaffolds of the BAT93-HiFi assembly. A dataset of 33,993 variants was retained after filtering by allele coincidence observed with BAT93-HiFi. Most of these variants (93.2%) were present in non-coding regions. The 6.8% variants in coding regions consisted of 1.7% silent mutations, 1.8% missense, 0.7% nonsense and 2.6% in prime UTR regions. A single mutation (A to T) was identified in BAT93-M822 in the *I* cluster spanning ~700 kb in BAT93-HiFi. This mutation is in the TNL gene, chr2g0069161, located near the proximal border of the TNL cluster (Fig. 2a). A Tm-shift marker assay of the mutation revealed perfect co-segregation with the resistance phenotype in 248 F_2_ individuals, confirming that chr2g0069161 was the *I* gene. Disregarding a 6.5 kb insertion (see below), the predicted coding sequence with a length of 3996 bp encodes a protein of 1332 amino acids (aa) with an N-terminal Toll/interleukin-1 receptor (TIR) domain, a central nucleotide-binding (NB-ARC) domain, a leucine-rich repeats domain (LRR) encompassing 23 predicted LRRs, and a C-terminal jelly roll/Ig-like domain (C-JID) (Fig. 2b; Supplementary Fig. 5). More precisely, the mutation in mutant BAT93-M822 is located in the splicing acceptor site of intron 1 and is predicted to lead to exon skipping (Fig. 2b). In agreement with this prediction, RNA Nanopore sequencing of BAT93-M822 mutant confirmed that chr2g0069161 undergoes an exon skipping leading to a putative 972 aa truncated protein (Fig. 2b, Supplementary Fig. 6).

Surprisingly, we observed a ~ 6.5 kb TE inserted in gene chr2g0069161 in BAT93-HiFi assembly (Fig. 2b). This insertion introduces a premature stop codon that is expected to result in a truncated protein lacking part of the LRR and C-JID domains, suggesting that this TNL gene is not functional. The remapped BAT93-HiFi reads (Supplementary Fig. 7) confirmed a reliable assembly for the region with many long HiFi reads covering gene chr2g0069161. Additional PCR experiments using primers amplifying a junction region between the TE and gene chr2g0069161, unambiguously validated presence of this TE in gene chr2g0069161 (Supplementary Fig. 8a). Furthermore, dot plot analysis of gene chr2g0069161 (from BAT93-HiFi) with either BAT93 BAC clones developed by our group in 2013^22^ or with contigs from another BAT93 assembly (BAT93-Cinestav) showed that this TE is only present in chr2g0069161 in BAT93-HiFi (Supplementary Fig. 9). Altogether, this led us to conclude that a TE inserted recently in BAT93 during seed propagation in our greenhouse. Consequently, we have a second independent mutant for gene chr2g0069161 in BAT93, referred to as BAT93-TE, corresponding to plants homozygous for the TE insertion in gene chr2g0069161. BAT93-TE inoculated with BCMNV-NL3 exhibited mosaic symptoms, demonstrating that the *I* gene is not functional in BAT93-TE. Moreover, 16 independent F_1_ plants from a cross between the two mutants (BAT93-M822/BAT93-TE) were susceptible after BCMNV infection, confirming that chr2g0069161 corresponds to the *I* gene. In agreement with the phenotypic data, quantification of the BCMNV-NL3 virus titer by RT-qPCR in systemic leaves 11 days post-infection (dpi) showed a significantly higher viral RNA titer in both mutants compared to BAT93-WT. BAT93-WT exhibited a low virus titer, significantly different from the mock inoculation, indicating that BCMNV was transported systemically (Fig. 3b). Transversal stem cuts from BAT93-WT after infection with BCMNV showed necrotic phloem tissues, while healthy phloem was observed in both mutants (Supplementary Fig. 10). Finally, comparative sequence analysis confirms *chr2g0069161* as the *I* gene, shareing 100% sequence identity with TNL *Pv5-593.02G316500* in genotype 5-593 (*I* genotype), while no clear homolog exists in *ii* genotypes G19833, JaloEEP558, Labor Ovalle and UI111 (Fig. 2).

Furthermore, the isolation of the *I* gene allowed us to design an *I*-based specific marker. This PCR-based marker, referred to as I-149, produced a short amplification product (149 bp) specific to the *I* gene in BAT93-WT (Supplemental Fig. 8b), since no amplification product was observed in BAT93-TE, despite the presence of the 31 remaining TNL from the *I* cluster.

### The *I* gene confers a broad-spectrum resistance against potyviruses but not comoviruses

To determine if the *I* gene confers resistance to other potyviruses, the two mutants (BAT93-TE and BAT93-M822) were tested with BCMV, WMV and ZYMV. After infection with BCMV-RU1 at 21 dpi, the resistant genotype BAT93-WT showed no symptoms on systemic leaves, whereas the two independent mutants BAT93-TE and BAT93-M822 exhibited characteristic mosaic symptoms on systemic leaves (Fig. 3c). Susceptibility of the two mutants was confirmed by significantly higher viral RNA titer of BCMV-RU1 detected by RT-qPCR in systemic leaves compared to BAT93-WT and Mock plants (Fig. 3d). The two *I* mutants were also susceptible to the US-6 strain of BCMV (Supplementary Fig. 11). Unlike BAT93-WT, both BAT93-TE and BAT93-M822 developed chloronecrotic lesions on the inoculated leaves after infection with WMV and ZYMV. This demonstrates that the extreme resistance response of BAT93-WT to these potyviruses is due to the *I* gene, although the lack of systemic infection, particularly for ZYMV, indicates additional resistance determinants in the BAT93 genome, likely acting at distinct infection stages (Supplementary Fig. 12).

*R-BPMV* gene, conferring resistance to BPMV, a comovirus, was also described as mapping to the *I* cluster^22,29^. To test whether the *I* gene also confers resistance to BPMV, both mutants were inoculated with BPMV (Fig. 3e). The same local HR was observed on inoculated leaves of BAT93-WT, BAT93-TE and BAT93-M822, while the susceptible control, Black Valentine (BV), showed mosaic symptoms. Consequently, despite their mutations in the *I* gene, both mutants remained resistant to BPMV, demonstrating that *I* and *R-BPMV* correspond to two different *R* genes within the same TNL cluster.

### Characterization of the TE inserted in the *I* gene

**Fig. 4:**
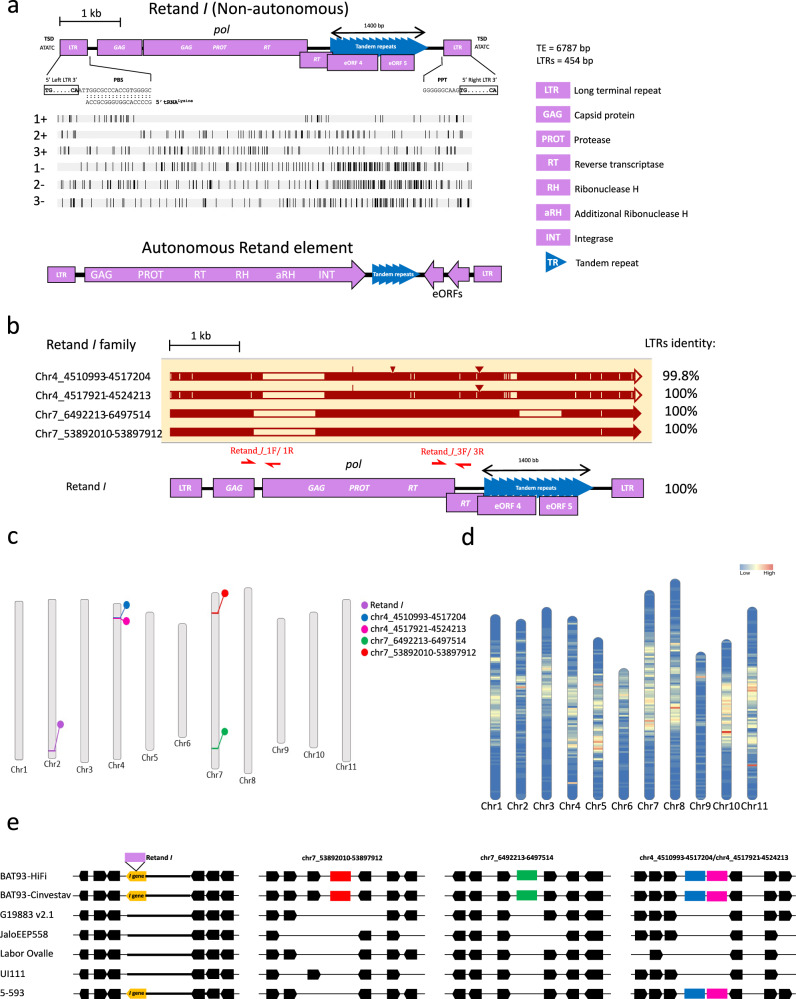
Characterization of the Retand *I* family in BAT93-HiFi assembly. **a** Scheme of the non-autonomous Retand *I* and autonomous Retand elements with structural and functional features. *LTR* long terminal repeat (starting with 5’-TG-3’ and ending with 5’-CA-3’); *PBS* primer binding site (its complementarity to the 3’ end of tRNA-Lysine is indicated); *PPT* polypurine tract; *TSD* target site duplication. Positions of stop codons are plotted in three sense and three anti-sense reading frames. Each vertical line represents a single stop codon present in the respective frame. **b** Schematic alignment of the 4 elements homologous to Retand *I* found by BLASTn in BAT93-HiFi genome assembly (the Retand *I* family), compared to the structure of Retand *I*. White spaces represent deletions and red triangles represent insertions in the homologous elements. Red arrows correspond to PCR primers that specifically amplify the Retand *I* element (Retand_*I*_1F/1 R, Supplementary Table 8) or that amplify the five elements of the Retand *I* family (Retand_*I*_3F/3 R, Supplementary Table 8). **c** Chromosomal distribution of the five transposable elements constituting the Retand *I* family in the BAT93-HiFi genome assembly. **d** Chromosomal distribution and density of the 3034 Retand elements (autonomous and non-autonomous elements) along the pseudomolecules of BAT93-HiFi genome assembly. **e** Synteny analysis of the five Retand *I* family elements in different *P. vulgaris* genome assemblies. Colored rectangles and black arrows correspond to the different Retand elements of Retand *I* family and conserved annotated genes, respectively. Source data are provided as Source Data file.

Annotation of the ~6.5 kb sequence inserted in the *I* gene chr2g0069161 in BAT93-TE revealed that the TE is a class I LTR retrotransposon of the Ty3/Gypsy Retand lineage. This TE, referred to as Retand *I*, is 6787 bp long, and contains 100% identical long terminal repeats (LTRs) indicative of a very recent insertion event and features a primer-binding site (PBS) and a polypurine tract (PPT), essential for retrotransposon replication (Fig. 4a). Moreover, the Retand *I* element showed two copies of the retrotransposon Gag protein (GAG) across the six reading frames. The second GAG is associated with an aspartic proteinase (PROT) and a reverse transcriptase protein (RT) within a polyprotein. An additional RT domain is encoded in an overlapping reading-frame. Downstream to this RT, two extra ORFs of unknown function overlap with a ~ 1400 bp tandem repeat region containing 20 monomers of a 87 bp consensus sequence (Supplementary Fig. 13), exclusively found in *Phaseolus* genomes.

In contrast to autonomous Retand elements, which encode all proteins required for retrotransposition, Retand *I* lacks three essential proteins —a ribonuclease H (RH), an additional RH (aRH) and an Integrase (INT)—and is therefore classified as a non-autonomous Retand element^30^. A BLASTn search of BAT93-TE genome assembly, identified four additional highly similar (sequence identity > 90%) non-autonomous Retand elements, with two located on chromosome 7 and two closely linked on chromosome 4 (Fig. 4b). Together, these 5 sequences define the Retand *I* sub-family. While most of the 3034 Retand elements were concentrated in pericentromeric regions, the five members of the Retand *I* sub-family were distributed in distal chromosomal regions (Fig. 4c, d).

Synteny analysis revealed that, except for genotype 5-593, which harbors the two elements located on chromosome 4, the corresponding syntenic regions in the other four genotypes lacked Retand *I* family elements (Fig. 4e). By contrast, BAT93-HiFi and BAT93-Cinestav genome assemblies contain four and five elements of the Retand *I* sub-family, respectively. Notably, the Retand *I* inserted in the *I* gene is the only additional element present in BAT93-TE compared to BAT93-Cinestav. Altogether, these findings support the hypothesis of a recent amplification of Retand *I* sub-family in certain Mesoamerican genotypes and suggest that insertion of Retand *I* into the *I* gene is a recent and unique event.

### Repeats and TEs in *Phaseolus* genomes

To investigate whether the expansion of the Retand lineage is specific to common bean, the presence of Retand and other repeats was analyzed in high-quality genome assemblies of legumes and species closely related to *P. vulgaris*, *P. lunatus* and *P. acutifolius*^31,32^. In common bean BAT93-HiFi and JaloEEP558-HiFi as well as in common bean G19833 v2.1, the repeated sequences account for ~50 % of the genome (~315 Mb/~630 Mb) while in other legume genomes like pea (*Pisum sativum*) and soybean (*Glycine max*) they represent 77% (3010 Mb/3920 Mb) and 62% (605 Mb/978 Mb), respectively (Supplementary Fig. 14a). Among the well identified repeated sequences, the LTR/Ty3/Gypsy elements constitute the largest TE family in common bean genomes ~8-13%. This is also the case in pea and soybean, where LTR/Ty3/Gypsy elements account for 55% and 30% of the total genome, respectively (Supplementary Fig. 14b).

**Fig. 5:**
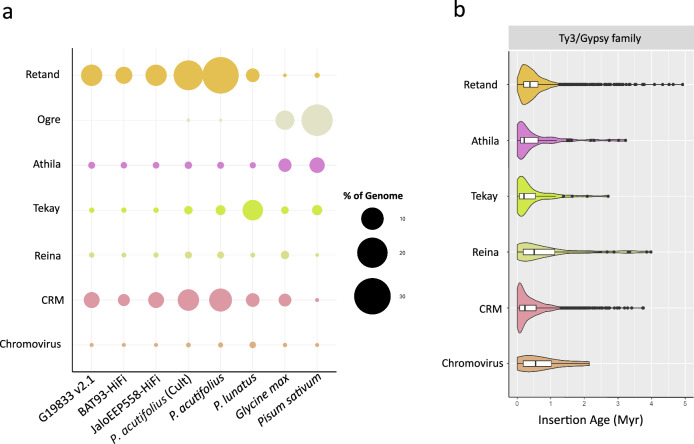
Repetitive sequence composition in *P. vulgaris* genome assemblies compared with other legume genomes. **a** Percentage of the genome assembly occupied by the Gypsy TE families in different *Phaseolus* genome assemblies compared with the soybean and pea genomes. **b** Age distribution of full-length elements long terminal repeat-retrotransposon (LTR-RT) Gypsy families in the BAT93-HiFi genome assembly. Box plots indicate the median (center line), with the 25th and 75th percentiles (bounds of box). Whiskers represent the minima and maxima (excluding outliers, shown as individual points). Violin shapes show the kernel density of the data. Source data are provided as Source Data file.

Further TEs classification showed that in common bean, Retand constitutes the major lineage of LTR/Ty3/Gypsy elements, representing ~15-25% of the TEs and ~5-9 % of the genome (Supplementary Fig. 14c; Supplementary Table 1) followed by the CMR elements, representing ~12–22 % of the TEs. By contrast, Retand elements are almost absent in pea and soybean, representing less than 1% of their genomes. In those species, Ogre TE family constitutes the major LTR/Ty3/Gypsy lineage accounting for 46% and 20% of the TEs, corresponding to 20% and 5% of pea and soybean genomes, respectively. The Retand lineage expanded in all *Phaseolus* species, occupying from 5% (in *P. lunatus*) to 30 % (in *P. acutifolius* wild) of the genome, whereas Ogre elements are completely absent except in *P. acutifolius* where few Ogre TE elements have been found (Fig. 5a). By contrast, for LTR/Ty1/Copia lineages, no notable differences were observed between legume genomes (*Phaseolus*, soybean, pea) (Supplementary Fig. 15). Insertion age estimates of LTR/Ty3/Gypsy lineages in BAT93-HiFi revealed that most of the Retand elements have recent insertion times (Median= 0.4 Myr), despite including a few ancient TEs members dating 5 Myr (Fig. 5b). Similar insertion age distributions of LTR/Gypsy lineages were observed in the genomes of G19833v2.1 and JaloEEP558-HiFi (Supplementary Fig. 16). Overall, these results suggest that the Retand family has recently expanded in the *Phaseolus* genus.

### Retand *I* activation

Activation of TEs have been observed under a variety of abiotic and biotic stresses^33^. To examine if transcription can be activated after stress, we designed primers specific for Retand *I* and for the Retand *I* sub-family (5 members), taking advantage of the fact that they present two RT domains allowing the design of specific primers for the Retand *I* sub-family (Retand_I_3F/3 R; Fig. 4b). After inoculation with BCMNV, transcription of Retand *I* and of Retand *I* sub-family were significantly induced at 5 dpi compared to mock plants (Supplementary Fig. 17). Consequently, the Retand *I* element inserted in the *I* gene and also the Retand *I* sub-family respond to the BCMNV biotic stress. Expression of *I* gene was slightly up-regulated at 7 hours post-inoculation (hpi) after BCMNV inoculation but this induction was not significant (Supplementary Fig. 17). Therefore, the *I* gene did not respond to the BCMNV stress at any of the evaluated time points and we could exclude that the observed activation of Retand *I* could be due the upregulation of the *I* gene (Supplementary Fig. 17).

## Discussion

The *I* gene in common bean is a source of durable and broad-spectrum resistance to potyviruses that has attracted long-standing interest from both the scientific community and plant breeders. Despite intense research efforts, *I* gene identification and cloning remained elusive until now. Leveraging high-quality long-read genome assemblies scaffolded with Hi-C, alongside two independent loss-of-function mutants (EMS-induced and TE-insertion), we identified the *I* gene and resolved the complex *I* cluster both molecularly and functionally, validating its role in potyvirus resistance. The EMS-induced and the TE-insertion mutants represent stable *NLR* loss-of-function lines in common bean, a species in which gene validation remains particularly challenging due to inefficient transformation protocols^34^. Both mutants displayed susceptibility to BCMV, BCMNV, ZYMV, and WMV, confirming that the *I* gene confers broad-spectrum resistance. Notably, the *I* gene mutants were resistant to BPMV, a comovirus, suggesting the presence of additional functional TNLs within the *I* gene cluster^22,29^.

We generated two chromosome-level assemblies of *P. vulgaris* BAT93 and JaloEEP558, the parental lines of a widely used RIL population which formed the basis of an important reference genetic linkage map in common bean^35^. These assemblies enabled us to better characterize the *I* gene cluster and represent a valuable resource for future breeding efforts and genomic studies. The *I* gene encodes a TNL protein containing a C-JID domain and is located within a highly dynamic resistance gene cluster, where the number of TNL genes varies dramatically from a single copy to as many as 34 across different genotypes. Similar structural variability has been observed in other plant *NLR* clusters, such as the *Rp1* locus in maize (*Zea mays*)^36^ and the *Pm69* cluster in wheat (*Triticum aestivum*)^37^. These variations often result from tandem gene duplications, frequently driven by unequal crossing-over, as well as intra-cluster chromosomal rearrangements and gene conversion events^5^. The high variability in TNL copy number observed among common bean genotypes underscores the rapid evolutionary dynamics of *NLR* gene clusters, even during relatively evolutionary-short timescales^38^. These results help to clarify the mechanism underlying the suppression of recombination at the *I* gene cluster, consistent with the known impact of structural variation on meiotic crossover formation^39^. The two parental lines of the RILs population, BAT93 (harboring 32 TNLs) and JaloEEP558 (with only a single TNL), exemplify this effect, as the pronounced structural divergence between their *I* clusters is associated with markedly reduced recombination^22^. Previous efforts to clone the *I* gene were hampered by this suppression and the repetitive nature of the *I* cluster^22,23^, but the use of high-quality genomic assemblies and mutants enabled isolation of *I*.

Our analyses revealed that *I*-carrying genotypes, BAT93 and 5-593, exhibit high sequence conservation at the *I* locus and contain a large number of TNL genes (>32) (Fig. 2; Supplementary Fig. 3). By contrast, *ii* genotypes display substantially greater structural variability and possess markedly fewer TNLs at the *I* cluster, consistent with early RFLP-based mapping results^23^. We propose that the *I* gene originated through a segmental duplication (or intracluster chromosomal rearrangements) event leading to high number of TNL (over 30). This large TNL cluster was subsequently introgressed as an intact block into other cultivated *ii* genotypes due to suppressed recombination within the *I* cluster due to extreme structural variation. Although initially discovered in snap bean, the *I* gene has also been found in black bean landraces from Central America. In fact, the *I* gene in BAT93 is derived from such a landrace. However, whether the *I* gene originated post- or pre-domestication remains an open question.

Our analysis revealed that the Retand family is the most abundant TE family in the *P. vulgaris* genome, with the Retand *I* sub-family responsible for the natural insertion disrupting the *I* gene in BAT93-TE. The presence of this Retand *I* element in BAT93-HiFi but not in BAT93-Cinvestav, nor in the corresponding BAT93 BAC clone, confirms its recent insertion, during seed propagation in our greenhouse. While LTR retrotransposon bursts are often invoked to explain TE-driven evolution^40^, the Retand *I* insertion appears to be a sporadic event, as it is the only additional Retand *I* sub-family element detected in BAT93-HiFi compared to BAT93-Cinvestav. Importantly, Retand *I* subfamily elements were transcriptionally activated under biotic stress (BCMNV infection), including the element inserted in *I*, highlighting stress-induced TE mobility. Such activation echoes findings in other species, for instance *Bs1* in maize^41^ and *Tnt1* in tobacco^42^, suggesting a conserved mechanism by which LTR retrotransposons contribute to genome plasticity under stress.

TEs are important and dynamic components of plant genomes highly diverse in terms of abundance across plant species^43^. Comparative analysis with other legumes revealed that while Ogre elements dominate genome size variation in pea, soybean, and faba bean^44^, *Phaseolus* genomes are instead enriched in Retand elements^45^ mostly in pericentromeric regions. By contrast, *Retand* elements have a subtelomeric distribution in *Silene latifolia*, the species in which this group of Ty3/gypsy retrotransposons was recently discovered^46^, suggesting that these elements are predominantly accumulated in heterochromatin, mainly subtelomeric in *Silene* and pericentromeric in *Phaseolus*. Retand and Ogre belong to the OTA superclade of Ty3/gypsy retrotransposons and share structural features, including tandem repeats that may contribute to satellite repeat evolution (Supplementary Fig. 13)^46^. Further exploration of these relationships could illuminate how TE lineages diverge and specialize across legume genomes.

Our results also provide insights into black root disease that has puzzled breeders for decades. Black root, characterized by systemic necrosis, was initially described as a new virus disease affecting snap beans^47^. Much later, this systemic necrosis was hypothesized to result from incomplete resistance mediated by the *I* gene^48^. Taken together, our results indicate that the systemic necrosis response, induced by BCMNV in *I* bearing genotypes, reflects an incompatible host–pathogen defense response mediated by the *I* gene. Indeed, in the two *I* mutants (BAT93-TE and BAT93-M822) this systemic necrosis is abolished, confirming that this response is dependent on the *I* gene (Fig. 3a). It has been proposed that systemic necrosis may result from an inefficient HR, leading to a systemic HR due to the virus escaping the primary infection site^49^. In agreement with that, we detected BCMNV in BAT93-WT uninfected leaves undergoing systemic necrosis (Fig. 3b).

In addition to providing functional and evolutionary insights into the *I* gene cluster, this study also supplies *I*-based specific markers that enable precise selection of the gene, thereby accelerating its deployment in bean breeding programs. In particular, the molecular marker (I-149) would benefit breeding efforts focused on pyramiding the dominant *I* gene with recessive genes, such as *bc-u*^d^*, bc-u*^r^*, bc-1*, *bc-2*, and *bc-3*, in order to prevent the detrimental systemic necrosis observed with BCMNV in *I* bearing genotypes^50^. This strategy is crucial in Central and East Africa, where BCMNV is endemic^51^. While molecular markers are already available for most of these recessive genes, our study provides marker within the *I* gene itself, preventing recombination between the marker and the target gene.

Furthermore, the molecular identification of the *I* gene opens avenues for targeted engineering to enhance its effectiveness. A mutant form of the potato *Rx* gene, known as RxM1, confers strong resistance to *Potato virus X* (PVX) and triggers systemic necrosis in response to another potexvirus, *Poplar mosaic virus* (PopMV) a reaction analogous to the response of *I-*gene-containing bean plants to BCMNV^52^. Interestingly, Harris et al.^53^ demonstrated that further mutagenesis of the RxM1 sequence, introducing change in the amino acid composition of the nucleotide-binding pocket, resulted in novel immune receptors capable of conferring resistance to both PopMV and PVX. Similar approaches could improve *I*-mediated resistance by preventing the onset of systemic necrosis in response to BCMNV. Recent CRISPR-based genome editing, utilizing viral delivery of an RNA-guided genome editor, offer an attractive transgenic-free strategy to achieve this goal^54^.

Our work also illustrates how natural, spontaneous mutations during seed propagation can be revealed through genome resequencing. With the advent of long-read sequencing, the study of spontaneous mutation is shifting from single-gene perspectives to genome-wide resolution^55^, paving the way for broader discoveries in plant evolution and breeding. More broadly, this work illustrates how combining long-read assemblies with spontaneous or induced mutants can resolve long-standing questions in plant disease resistance and genome evolution.

## Methods

### Plant and virus materials

BAT93 is a Mesoamerican breeding line, developed at the Centro International de Agricultura Tropical (CIAT) and derived from a cross involving four Middle American genotypes (Veranic 2, Tlalnepantla 64, Jamapa, Tara). BAT93 seeds were a gift from Paul Gepts (UC Davis; USA) to VG in 1994. Since 1994, BAT93 seeds have been annually multiplicated in IPS2 greenhouse (Gif sur Yvette, France). BAT93-TE is a natural mutant of BAT93 resulting from the insertion of a Retand transposable element in the *I* gene during seed multiplication in Gif sur Yvette (France). BAT93-TE is homozygous for the TE insertion. M822 is an M3 EMS BAT93 line presenting a mutation in the *I* gene that was identified from the screening of 1657 M3 EMS BAT93 lines developed by Porch et al.^28^. JaloEEP558 is a selection from the Andean landrace Jalo obtained at the Estação Experimental de Pato de Minas (Minas Gerais, Brazil). Six viral strains were used in this study: BCMNV isolate NL3 (BCMNV-NL3), BCMV isolate US-6 (BCMV-US-6)^56^, BCMV isolate RU1 (BCMV-RU1)^57^, BPMV isolate IA-Di1^29^, WMV isolate C05-270^58^ and ZYMV isolate E15-PAT^59^.

### DNA extraction and sequencing

*P. vulgaris* BAT93 and JaloEEP558 were grown in a chamber at 23 °C under long-day conditions (16 h day/8 h night). Young trifoliate leaves were collected from 10-days-old plants after 3 days of dark treatment to optimize DNA extraction, and flash-frozen in liquid nitrogen. High molecular weight (HMW) DNA was extracted and sequenced with a PacBio Sequel II system by GENTYANE platform (Clermont-Ferrand, France). In total, 21.09 Gb of PacBio reads with 31x physical coverage, and 34,7 Gb of PacBio reads with 57x physical coverage were generated for BAT93 and JaloEEPP558, respectively (Supplementary Table 2).

### Reference genomes

Genome assemblies of G19833 (v2.1), Labor Ovalle (v1.1), UI111 (v1.1), 5-593 (v1.1), *P. acutifolius* (v1.0 and WLD v2.0), *P. lunatus* (v1), *Glycine max* (v2.0), and *Pisum sativum* (v1a) were retrieved from Phytozome (v13) (https://phytozome-next.jgi.doe.gov)^60^. The genome assembly of BAT93-Cinvestav was retrieved from the Cinvestav data repository (http://mazorka.langebio.cinvestav.mx).

### De novo assembly

Contigs-level genome assemblies of BAT93 and JaloEEP558^25^ were further assembled into chromosome-scale pseudomolecules using Hi-C scaffolding. Hi-C scaffolding was performed using Juicer pipeline (v1.5.7)^61^ followed by a 3D-DNA pipeline (v3d-dna-529ccf4)^62^. Oriented contigs along each chromosome were separated by 1000 Ns. Hi-C reads were realigned to scaffolds and contigs with bwa v0.7.17 (default parameters), converted to BAM with samtools sort v1.10, and indexed. The alignment file was then inspected to detect pairs of sequences with elevated interaction frequency at their ends. When visual inspection of the Hi-C contact map suggested a high probability of co-location, the contig was manually placed on the corresponding side of the scaffold, and such manually placed contigs were separated by 500 Ns (Supplementary Data 1 and 2). The resulting assemblies were polished using the Juicebox Assembly Tools (v 1.11.08)^63^. Final assemblies (namely BAT93-HiFi and JaloEEP558-HiFi) contain eleven chromosome-scale pseudomolecules with a total length of 569.4 and 542.4 Mb for BAT93 and JaloEEP558, respectively (Table 1 and Supplementary Table 3). The first 100 biggest unplaced scaffolds were inspected for bean specific subtelomeric repeats “khipu”^64^, centromeric repeats Nazca and CentPv2^65^, and the canonic (TTTAGGG) plant telomeric repeats^66^. Presence of repeats and counts of Hi-C links were used to manually anchor additional unplaced scaffolds. Chromosomes were numbered and oriented using the reference assembly of G19833 v2.1.

### In situ Hi-C assay

Trifoliate leaves of 10-days-old plants from the common bean genotypes BAT93 and JaloEEP558 were used to perform the in situ Hi-C experiment according to Concia et al.^67^ using *Dpn*II enzyme (New England Biolabs (NEB), Ipswich, MA, USA). DNA libraries were prepared using NEBNext Ultra II DNA library preparation kit (NEB) according to the manufacturer’s instructions (9 cycles for the PCR amplification step). DNA libraries were checked for quality and quantified using a 2100 Bioanalyzer (Agilent) and the libraries were subjected to 2 × 75 bp high-throughput sequencing by NextSeq 500 (Illumina) (Supplementary Table 4).

### Circos plot

Genome assemblies of BAT93-HiFi and JaloEEP558-HiFi were divided in 50 kb bins using BEDTools (v2.28.0)^68^. Genome scale visualizations were generated using Circos software (v0.69.8) (http://circos.ca/)^69^.

### Synteny analysis

Whole genome alignment dot plots between assemblies of G19833 v2.1, BAT93-HiFi, JaloEEP558-HiFi and phasIbeam10.0 were performed using D-Genies with the Mashmap algorithm (v2.0) and with default parameters^70^. Syntenic collinear blocks between genome assemblies were identified using MCScan v2^71^. Collinear blocks were visualized using the python JCVI utilities libraries (https://github.com/tanghaibao/jcvi). Micro-synteny analysis was performed by identifying orthologous pairs of best reciprocal BLASTp hits (e-value cutoff = 10^-10^) between the genome assemblies of BAT93-HiFi, JaloEEP558-HiFi, G19833 v2.1, Labor Ovalle, UI111 and 5-593 using Crb-BLAST^72^.

### Annotation of protein-coding genes and lncRNAs

For the transcriptome-based gene prediction, RNA-seq data from BAT93 was retrieved from the ENA data repository PRJNA221782^27^ and from Alvarez-Diaz et al.^73^ Transcriptomic data from JaloEEP558 was recovered from the ENA repository PRJNA604533^74^. For JaloEEP558, additional RNA-seq data was generated from different tissues (cotyledonary and trifoliate leaves, roots and stem) (Supplementary Table 5 and 6).

For each RNA-seq library, transcripts were predicted using a Snakemake pipeline^75^. Briefly, RNA-seq quality was assessed with fastQC screen^76^ and adapters and low-quality bases were trimmed with Cutadapt^77^. Filtered reads were mapped to the BAT93-HiFi and JaloEEP558-HiFi genome assemblies respectively with STAR (with parameters: --outSAMtype BAM SortedByCoordinate --outSAMstrandField intronMotif --outFilterType BySJout --outSJfilterCountUniqueMin 3 2 2 2 --outSJfilterCountTotalMin 3 2 2 2)^78^ and transcripts predicted with StringTie pipeline (with parameters: --conservative -a 5 -g -3)^79^. We chose to predict transcripts on strand + and – separately and to merge prediction with StringTie merge (with parameters: “-F 0.3”).

Annotation of protein-coding genes and lncRNAs were predicted with a fully automated and parallelized egn-ep pipeline that carries out probabilistic sequence model training, genome masking, transcript and protein alignment computation and integrative gene modeling in EuGene software (release 4.2b)^80^. We used a 2 step-prediction, as we predicted gene model on each strand before merging annotation. For a reference protein database, we used databases of manually curated NLR proteins (G19833 v2.1), of *P. vulgaris* proteins curated for TE, and the uniprot database for Arabidopsis proteins with evidence at protein, transcript and homology levels.

### Functional annotation of protein-coding genes

Protein-coding genes were annotated through integration of different databases (https://lipm-gitlab.toulouse.inra.fr/LIPM-BIOINFO/nextflow-functionnalannotation). Predicted genes were functionally annotated by performing a BLASTp search against the UniProtKB Viridiplantae database and the NCBI non-redundant protein database with an e-value threshold of 1e-10. In addition, a comprehensive annotation was also achieved using InterProScan (v5.31–70.0)^81^, which includes motifs/domains prediction, functional classifications, protein family identification, transmembrane topology, predicted signal peptides and GO annotations. KAAS^82^ and KOBAS 3.0^83^ were used to search the KEGG GENES database for KO (KEGG Orthology) assignments and generating a KEGG pathway membership. PlantTFcat^84^ was also used to systematically analyze InterProScan domain and categorize possible chromatin regulators (CRs), transcription factors (TFs) and other transcriptional regulators (TRs) in the current assembly. In parallel, to complete the previous annotation, we performed a second search run using EggNOG database, a hierarchical, functionally and phylogenetically annotated orthology resource based on 5090 organisms and 2502 viruses^85^. All GO, KO and KEGG functional custom annotation were merged. The completeness of the genome annotation was evaluated with BUSCOs (v3.0.2) using the Embryophyta database (embryophyta_odb9)^86^ (Supplementary Table 7).

### *NLR* gene annotation

Disease resistance genes on the BAT93-HiFi and JaloEEP558-HiFi assemblies were identified using *NLR* annotator v2.1^87^ which classifies *NLRs* into subclasses (e.g., *TNLs* based on TIR domain), and using MEME (v4.9.1)^88^ with default parameters. LRR were characterized for the *I* gene using LRR Predictor (V1.0) (https://lrrpredictor.biochim.ro/). *NLRs* located in the *I* cluster were manually annotated using a combination of gene model prediction from EuGene^89^, *NLR* annotator^87^, and RNA-seq data (short reads from different organs from publicly available database and generated in this study (Supplementary Table 5 and 6) and long reads generated in this study (Supplementary Table 8). Visualization of the various annotation features, as well as manual inspection and editing, were performed using ARTEMIS (v18.1.0)^90^.

### De novo transposable-element and repeat annotation

Repetitive sequences in the genome assemblies of BAT93-HiFi, JaloEEP558-HiFi and G19833 v2.1, were identified and characterized using RepeatMasker (v4.0.7)^91^ and TEs were annotated using the Extensive de novo TE annotator (EDTA) pipeline^92^. A curated library of TE families in common bean published by Gao et al.^93^ supplemented with a set of manually annotated Retand elements, was provided to EDTA in order to improve TE annotation (Supplementary Data 3). Parameters used to run the pipeline were: “EDTA.pl -genome Genome.fa -species others -step all -cds./Genome.cds.fa –curatedlib./Phaseolus.pvTE.V2.fa -evaluate 1 -threads 32”. TEs were classified in subfamilies and lineages, using the Domain based Annotation of Transposable Elements program (DANTE)^30,94^. This similarity-based classification is based on phylogenetic relationships as well as distinct sequence and structural features of the elements. Further calculation of metrics and data visualization were carried out with custom scripts in R (v4.1.2)^95^.

Retand elements were annotated by using a combination of the following software: DANTE^30^, the protein motif search database MOTIF, and SnapGene version 6.2.1 (snapgene.com). Tandem repeats were identified and characterized by using the genomic similarity tool (YASS)^96^ and Spectral Repeat finder software^97^ with default parameters. Furthermore, autonomous elements were defined following the Neumann’s classification^30^. Retand elements encoding at least one copy of each of the six proteins required for retrotransposition were classified as autonomous elements, otherwise they were classified as non-autonomous.

### Retand *I* family characterization and synteny analysis

To identify the closest homologous copies of Retand *I* in BAT93-HiFi, homology searches were performed with BLASTn using the complete nucleic sequence of Retand *I* as query. BLASTn parameters were: sequence identity ≥ 90% and coverage ≥ 90%. Significant found elements were analyzed by multiple sequence alignments with CLUSTAL Omega^98^ with default parameters. Syntenic analysis of Retand *I* homologous elements was done in BAT93-Cinvestav, G19833 v2.1, JaloEEP558, LaborOvalle, UI111 and 5-593 using BLASTn with the nucleic sequences of Retand *I* family elements and their conserved flanking genes comprising a region of ~100 kb from BAT93-HiFi, as queries. BLASTn parameters were: sequence identity ≥ 90% and coverage ≥ 90%.

### Validation of the presence of the Retand element in *I* gene

To determine the homozygous or heterozygous genotype by PCR, specific primers were designed in the *I* gene (Gene2_seq_F/LRR2exp_R and GT3/GT8) and in Retand *I* targeting the junctions generated by the insertion of the TE (GT7 and GT4, used with GT8 or GT3 respectively) (Supplementary Fig. 8 and Supplementary Table 8). The presence of the Retand in the *I* gene was detected by amplification of a PCR product with the GT3/GT4 and GT7/GT8 pairs, and by absence of a PCR product with the GT3/GT8 and Gene2_seq_F/LRRexp_R pairs. The GeneI_4.3 F/GeneI_4R pair is specific to the *I* gene, leading to a 149bp-amplicon in BAT93-WT. PCRs were performed with the following thermal profile: initial denaturation at 94 °C for 5 min, followed by 30 cycles of denaturation at 94 °C for 30 s, annealing at 62 °C for 30 s, and extension at 72 °C for 1 min 30 s (for pairs GT3/GT4; GT7/GT8; GT3/GT8 and GeneI_4.3 F/GeneI_4R) and for 5 min (for pair Gene2_seq_F/LRR2exp_R). A final extension step was performed at 72 °C for 7 min. The amplification results were observed by electrophoresis on a 2% agarose gel.

### Genome-wide distribution of Retand elements

Genomic distribution of annotated Retand elements (autonomous and non-autonomous) including the Retand *I* family were plotted using the RIdeogram package^99^ in R version 4.3.1. (R Core Team 2016). The genomic distribution of autonomous and non-autonomous Retand elements were plotted by using the Phenogram Plot software with default parameters^100^.

### Estimation of insertion time for full length LTR-RTs

To estimate the insertion time of LTR retrotransposons (LTR-RT), the 5’- and 3’- LTR sequences for each full-length LTR-RT were aligned with MUSCLE v3.8.1^101^ with default parameters and used to calculate Kimura’s 2 parameter distances^102^. The insertion time (*T*) was determined with the below formula^103^:

1 $$T=K/2r$$

where *K* is the average number of substitutions per aligned site and *r* is an average substitution rate. We used the average substitution rate of 1.3 ×10^-8^ substitutions per synonymous site per year to calibrate the insertion times^104^.

### Sequencing of BAT93-M822 EMS mutant M3 line and variant calling

DNA of the *I* EMS mutant M3 line BAT93-M822 from the EMS BAT93 population with mutant phenotype for the *I* gene was extracted from 200 mg of leaf tissue collected from a single plant using QIAGEN DNeasy Plant Pro Kit (QIAGEN, Hilden, Germany). DNA concentration was quantified with NanoDrop 2000 (Thermo Fisher Scientific, Waltham, MA) and the DNA quality was evaluated by electrophoresis and visualization on a 1% agarose gel. DNA was sent to ‘Beijing Genomics Institute’ (BGI, Hongkong Tech Solution NGS Lab) for library construction and whole genome sequencing (WGS) of 150 bp paired-end reads to 24x mean coverage with a median insert size of 342 bp using DNBs (DNA nanoballs) platform. The sequence reads were aligned to the BAT93-HiFi reference genome using the Next Generation Sequencing Experience Platform (NGSEP v4.0.2) command “ReadsAligner”^105,106^ and a sorted BAM file was obtained using Samtools v1.9^107^. SNPs, small indels, and CNVs were discovered executing the NGSEP pipeline^108^, using parameters of maximum base quality score set to 30, minimum quality for reporting a variant to 40, heterozygosity rate to 0.0001 and maximum number of alignments allowed to start at the same reference site to 2. Finally, a VCF file was generated containing the called variants and each variant was annotated using the NGSEP command “VCFAnnotate”.

### Co-segregation test between the *I* resistance phenotype and the mutation in the *I* gene

F_2_ seeds from reciprocal crosses between BAT93-WT and the BAT93-M822 mutant [81 seeds for BAT93-WT/M822 (B x M) and 167 seeds for M822/BAT93-WT (M x B)] were planted in the greenhouse facility of U.S. Department of Agriculture (USDA-ARS), Washington, USA. The primary leaves of 10-days-old plants were inoculated with BCMV-US-6 using the mechanical rub-inoculation method (see below). Symptoms were recorded at 7-day intervals for four weeks. They included systemic mosaic (susceptible reaction – no functional *I* gene) or no symptoms (extreme resistance; presence of a functional *I* gene).

Leaf disc samples from trifoliolate leaves of each F_2_ plant at about 20 days after sowing were used for DNA extraction. F_1_ seeds from the reciprocal crosses BAT93-M822/BAT93-TE (M x TE) 7 seeds and BAT93-TE/BAT93-M822 (TE x M) 9 seeds were similarly inoculated with BCMNV-NL3.

Tm-shift marker assay for the causal SNP in gene chr2g0069161 (*I* gene) of BAT93-M822 was conducted (Supplementary Fig. 18). Allele-specific primers were generated for the polymorphic variant detected in BAT93-M822. Two forward primers with differing lengths of GC tails attached to their 5’ ends (M822v2_Fa and M822v2_Fb, Supplementary Table 9) and a common reverse primer (M822v2_R, Supplementary Table 9) were designed using the Primer3 software^109,110^ and according to the melting temperature (Tm)-shift SNP genotyping method developed by Wang et al.^111^. Tm-shift SNP markers were amplified by PCR using an Eppendorf Mastercycler (Eppendorf AG, Hamburg, Germany). The PCR volume was 20 μL, which included 20 ng of genomic DNA, 1X Taq buffer, 1.5 mM of MgCl_2_, 0.2 mM of dNTPs mix (Promega), 0.15 μM of each primer (two allele-specific forward primers and the common reverse primer), 1X EvaGreen® dye, and 0.1 μL of Taq polymerase (Promega). The PCR was performed with the following thermal profile: initial denaturation at 94 °C for 2 min, followed by 38 cycles of denaturation at 92 °C for 20 s, annealing at 60 °C for 20 s, and extension at 72 °C for 20 s. A final extension step was performed at 72 °C for 5 minutes. Melting point analysis for allele determination of the PCR products was conducted using a fluorescence-detecting QuantStudio 5 Real-Time PCR (Thermo Fisher Scientific, Waltham, MA, USA). EvaGreen fluorescent detection was performed for 15 s at 95 °C, and the melting curve data were collected with a ramp rate of 0.05 °C/s from 65 to 95 °C.

### Viral inoculation

The screening of the 1657 M3 BAT93 EMS lines was performed in USDA-ARS greenhouse by inoculating NL3 strain of BCMNV (BCMNV-NL3) and looking for systemic mosaic-mottled leaf symptoms^112^. Briefly, 10-days-old plants of each genotype were inoculated mechanically on their two primary leaves^112^. A Viral inoculum was prepared by grinding fresh leaves of infected source plants, squeezing through cheesecloth, diluting 1:10, and adding 500-mesh Carborundum powder prior to rub-inoculation^48^. Plants from *P. vulgaris* genotypes BAT93-WT (without Retand in the *I* gene), BAT93-TE (with Retand in the *I* gene) and BAT93-M822 (EMS mutant in the *I* gene) were grown in a growth chamber at 23 °C under long-day conditions (16 h-day/8 h-night). Six 9-days-old plants were inoculated with BCMV-RU-1, BCMV-US-6, BCMNV-NL3 and BPMV (strain IA-Di1) according to a viral rub-inoculation protocol adapted for BPMV^29,113^. Briefly, a viral inoculum was prepared by grinding frozen or fresh infected leaves from *P. vulgaris* cv. JaloEEP558 (for BCMV and BCMNV) or cv. Black Valentine (for BPMV) with a mortar and pestle in presence of mock buffer (50 mM potassium phosphate buffer, pH 7)^113^. Mechanical inoculation was then performed on one primary leaf of a healthy plant, using 0.037 mm Carborundum as an abrasive^113^. Mock plants were inoculated with pure mock buffer using the same procedure. Virus-inoculated plants were then placed in a growth chamber at 20 °C under long-day conditions (16 h-day/8 h-night), except for BPMV-infected plants, which were placed at 26 °C. The experiment was repeated twice.

Six 11-days-old plantlets of BAT93-WT, BAT93-TE and BAT93-M822 grown in an insect-proof greenhouse were rub-inoculated on the first expanded leaves with sap extracts of zucchini squash leaves infected with WMV isolate C05-270 and ZYMV E15-PAT. Briefly, a viral inoculum was prepared by grinding infected leaves with 0.03 M Na_2_HPO_4_ containing 0.2 % Na-diethyldithiocarbamate (DIECA) (1:10; w/v) using a mortar and pestle. Extracted juice was mixed with 400-mesh Carborundum (75 mg/mL) and activated charcoal (75 mg/mL) before rub-inoculation^114^. The experiment was repeated twice. For the second experiment, the plants were placed in a growth chamber at 23 °C/20 °C under long-day conditions (16 h-day/8 h-night).

### Observation of symptoms and virus titer

Symptom observations were performed on the inoculated leaves and at the plant apex at 2 weeks and 4 weeks, respectively after WMV and ZYMV inoculation. Symptoms were observed on systemic leaves at 11 dpi for BCMNV-NL3 inoculated plants and at 21 dpi for BCMV-RU-1 and BCMV-US-6 inoculated plants. Concurrently, the systemic leaves were sampled to establish the virus titer using a method based on RT-qPCR analysis by determining the amount of BCMV-RU1 and BCMNV-NL3 corresponding to the RNA fragment encoding the viral capsid protein and the plant Insulin-degrading enzyme gene (*PvIDE*, *Phvul.001G133200*) mRNA, to obtain a ratio of viral RNA to plant RNA. The following primers were used in the RT-qPCR: CPF02F/CPF02R for BCMV, BCMNV_3F/BCMNV_3R for BCMNV and IDE qPCR F/IDE qPCR R for *IDE* (for sequence of primers see Supplementary Table 9). Relative gene expression was then calculated using the 2^−∆Ct^ method on six biological replicates and three technical replicates.

### Expression of *I* gene and Retand elements after biotic stress

To perform a time-course analysis of expression of the *I* gene and Retand *I* family elements, inoculated (and mock) leaves were sampled at 0, 7, 24, 48 hpi and 5 dpi after infection with BCMNV-NL3 and expression was quantified by RT-qPCR using the specific primers listed in Supplementary Table 9 and depicted in Fig. 4b and in Supplementary Fig. 8: GeneI_5UTR_2F/GeneI_x1_2R are specific of the *I* gene, Retand_I_1F/Retand_I_1R are specific of the Retand *I* whereas Retand_I_3F/Retand_I_3R amplify the 5 Retand elements of the Retand *I* sub*-*family. To normalize gene expression, *PvIDE* was used as a reference. To calibrate gene expression on infected plants, gene expression in mock treatment was used for each gene and each time point. Relative gene expression in inoculated leaves compared with mock was calculated using the 2^−∆∆Ct^ method on three biological replicates and three technical replicates.

### RNA extraction and RT-qPCR analysis

Total RNA was extracted using the NucleoSpin RNA Plus Kit (Macherey-Nagel, Hœrdt, France). The RNA quantity and purity were determined on a NanoDrop 8000 spectrophotometer (Thermo Fisher Scientific, Waltham, USA) and RNA integrity was checked by electrophoresis on a 1% agarose gel. cDNA was synthesized from 1 μg of RNA using the ImProm-II™ Reverse Transcription System (Promega, Madison, WI, USA) according to the manufacturer’s protocol. Quantitative RT-PCR was performed using a LightCycler® 96 Instrument in a 15 μL volume reaction containing 5 μL of cDNA diluted 1:5, each specific primer with a final concentration of 0.01 μM each, 7.5 μL SYBR green premix (LightCycler® 480 SYBR Green Master, Roche Diagnostics, Meylan, France) and distilled water. Results were analyzed by using the software LightCycler® 96 version 1.1. Specific primer-pairs for the different targets were designed using Primer3 (v0.4.0) and checked for their specificity on BAT93-HiFi assembly using Primer-BLAST^115^.

### Histological analysis

The stem sections of mock and BCMNV-NL3 infected plants (17 dpi) were prepared by cutting the hypocotyl section, 1 cm below the cotyledonary node, and fixing them in 1% agarose blocks. Transverse cuts of 0.5 µm thickness were made using a Vibratome VTS1200S (Leica, USA). The cuts were rinsed with water and observed under an Olympus xc23 light microscope (Olympus, France).

### Statistical analysis

Statistical analysis was performed using GraphPad Prism version 8.0.1. (Boston, Massachusetts, USA, www.graphpad.com). Comparison between treatments was performed using the non-parametric Wilcoxon-Mann-Whitney U-test. Asterisks indicate the level of significance: * *p* < 0.05, ** *p* < 0.01 and *** *p* < 0.001.

### Reporting summary

Further information on research design is available in the Nature Portfolio Reporting Summary linked to this article.

## Supplementary information

Supplementary Information

Peer Review file

Description of Additional Supplementary Files

Supplementary Data 1

Supplementary Data 2

Supplementary Data 3

Supplementary Data 4

Reporting Summary

## Source data

Source Data

## Data Availability

The raw WGS reads of BAT93-M822 generated in this study have been deposited in the National Center of Biotechnology Information (NCBI) Sequence Read Archive (SRA) under BioProject PRJNA1042929 [https://www.ncbi.nlm.nih.gov/sra/SRX29143529]. The two high-quality genome assemblies for BAT93 and JaloEEP558 have been deposited in the NCBI under BioProject PRJNA1273100 and the corresponding annotations have deposited in French Government Data Research Repository [10.57745/JPMRAK]. The RNA-Seq and Hi-C data have been deposited in the NCBI SRA under BioProject PRJNA1273100. Source data are provided with this paper.
