# Supplementary material for: The I gene defines a dynamic NLR cluster conferring broad potyvirus resistance in common bean
Source: Nat Commun. 2026 May 30;17:7042. doi: 10.1038/s41467-026-73550-x (PMC13392006; doi:10.1038/s41467-026-73550-x)
Supplement: Supplementary file 1 — Supplementary Information [file 41467_2026_73550_MOESM1_ESM.pdf]

The *I* gene defines a dynamic *NLR* cluster conferring broad potyvirus resistance in common bean

Alvarez-Díaz and Soler-Garzón *et al.*

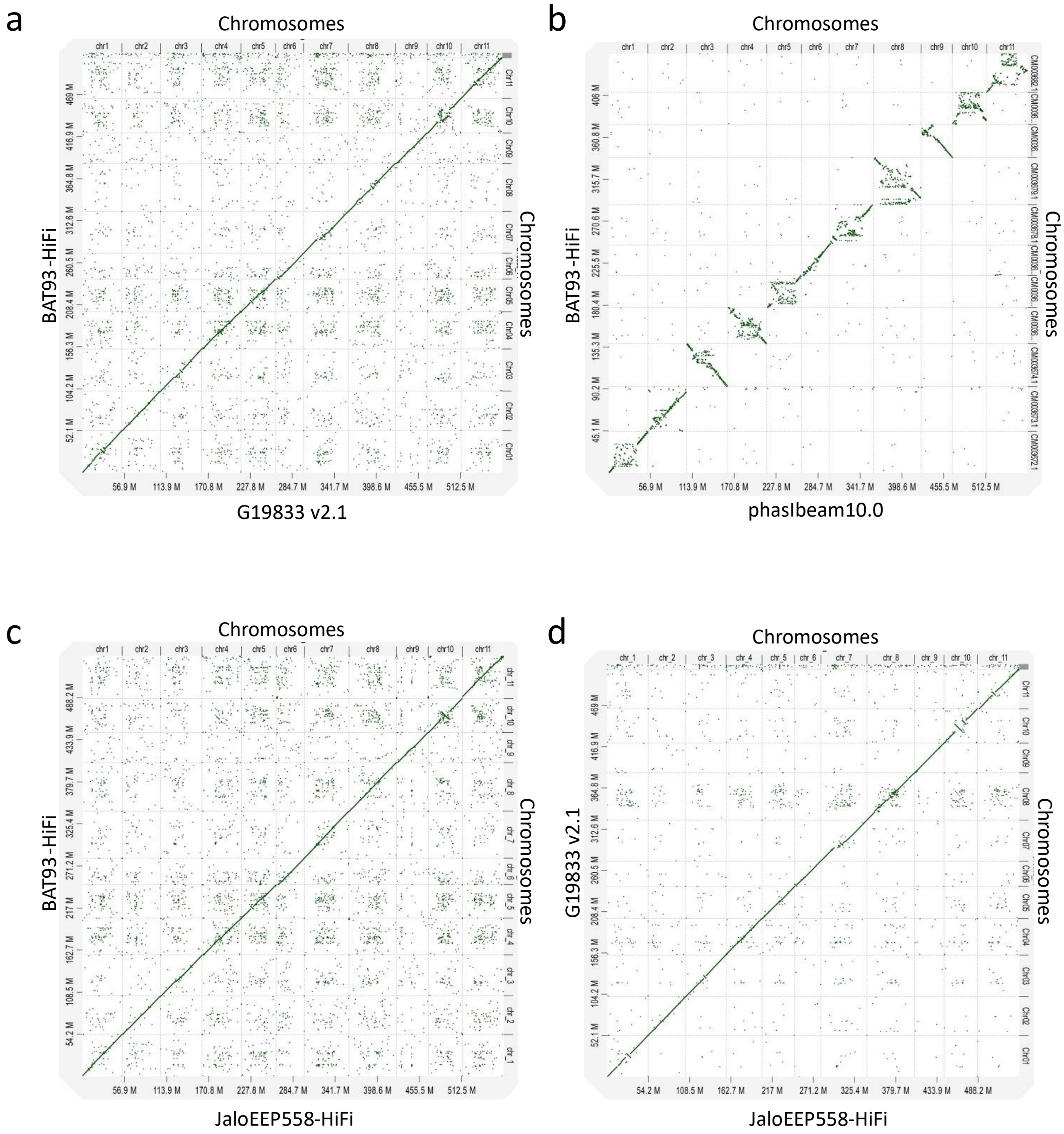

Supplementary Figure 1. Comparative synteny dot plot analysis. Analysis between assembled genomes of BAT93-HiFi with G19833 v2.1 (a), BAT93-HiFi with BAT93 (phasIbeam10.0) (b), BAT93-HiFi with JaloEEP558-HiFi (c) and G19833 v2.1 with JaloEEP558-HiFi (d).

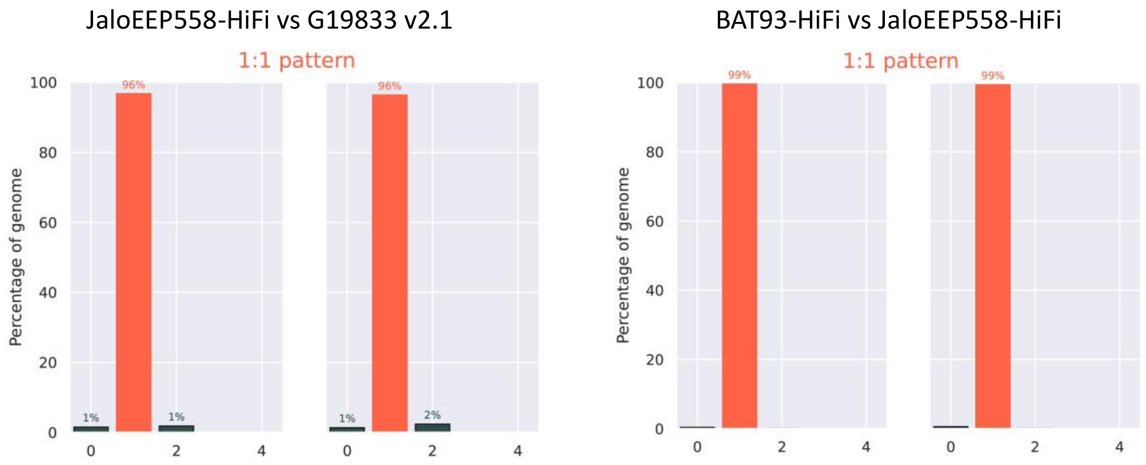

Supplementary Figure 2. Syntenic relationship between sequenced and reference genome assemblies.

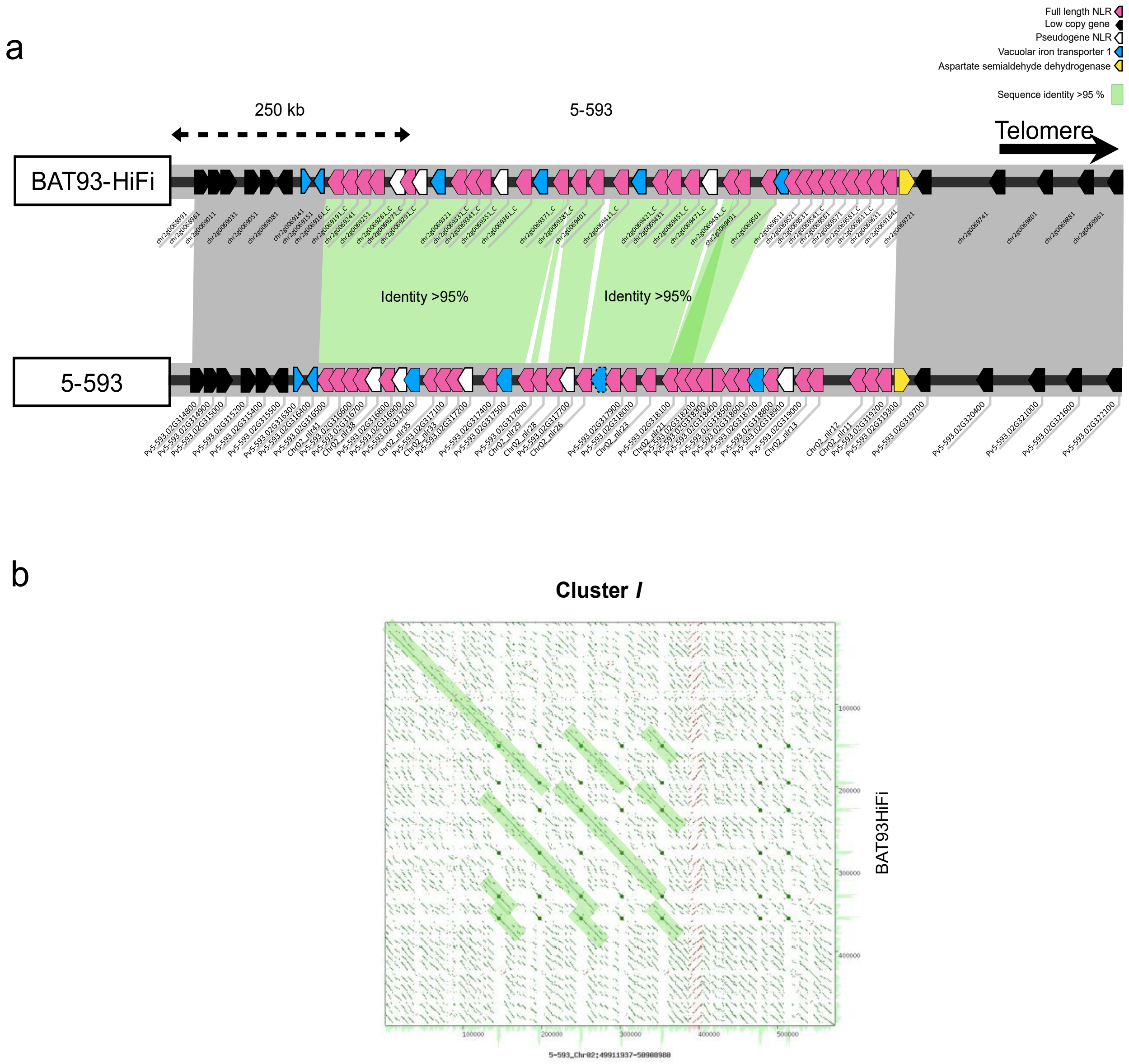

Supplementary Figure 3. Comparative analysis between two *I* bearing genotypes BAT93 and 5-593. a. Representation of the synteny of the *I* cluster. b. Dot-plot analysis of the *I* cluster between the two genotypes (TNL containing region).

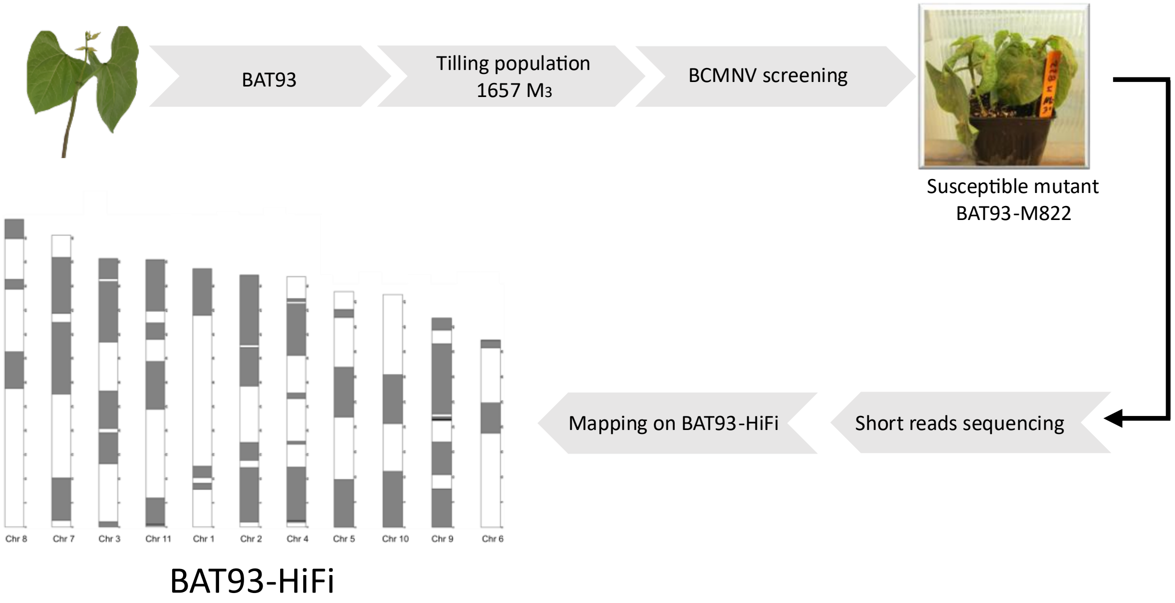

Supplementary Figure 4. Workflow for the identification of the *I* gene on the BAT93-HiFi genome assembly.

MSASSSSLAMSASLTKHDVFLSFRGEDTRDNFISHLRAALQRKNLQAYIDDRLVRGKEISDALLSAIEESKIYVVVF

TIR

SQNYASSTWCLNELTKILECKKRYGRDVIPVFYKVDPSTIRKQEERYKKAFDDHEQHFKHDMDKVQGWKDALTEAAG

P-loop

LSGWDSKVTRPENTLVEKIVEDILGKLNRYSISYDQGIIGIEKHIEGIQSLLHPESLDVRIIGICGMGGIGKSTICG

QIYHKLALQFDSSSLVLDVQDKIQRDGIDSIRTKYESEFLKEDKSSYLLYSNERLKRTKVLLILDDVTDSALLKRLI

NB-ARC

GGSDSFGQGSRIIMTSRDRQVLRNAGADDIYEVKELNFYDSLRLFSLHAFKQKSPEEFAYRELSVEVLRYAKGIPLA

LQILGSLLYGRTREAWESQLQKLEKGQHLDIFNVLKLSYDGLEEEEKNIFLDIACFFRGHEEIVVAETLADCGFSSK

Linker — LRR 1

IGIDILKDRGLISVFDGRIVMHDLIQEMGQEIVRKECPWPPGKRSRLFNAKEIFEVLSKNKGSDAIQCILLDTSKIK

LRR 2 — LRR 3 — LRR 4

KVKVHARTFEKMENLRMLMLYYSSPINESKVSLESPLVDLPDTLKILYWNDFPQRFWPPNFCPQNLVILEMPGCHLR

LRR 5 — LRR 6 — LRR 7

QLWEGDQNLPKLKRLNLSDSSNLTRIPDLSLSPNIEEIILSFCEKLIEVHSSIFLSKLNCLCLDHCYELKSVNIPSN

LRR 8

ILSTLPGLIILSFCRKLNMFSTSEPRFPHVKLERQRQTFSRSPHQKLGHQRGNFSRFPHQEPNIILQSASFVHIFPS

TE Insertion site — LRR 9 — LRR 10 — LRR 11

SSEIFSITFDRYNEEEVANNTVYLQFEVSANLRGGVPLNFQSLKNLCYLDLSDCSSLTIFPFDLSDMKFLKQLSLRG

LRR 12 — LRR 13 — LRR 14

CSKLENLPEIQDTLEDLAVLILDATAIQALPSSLCRLVGLQQLSLRRCFNLHIIPSSIGTLTRLCKLDLTHCNSLQT

LRR 15 — LRR 16 — LRR 17

FPSTIFNLKLRKLDFRGCSRLRSFPEITEPAHTFAHINLTCTAVKELPSSFGNLVNLRSLELQECIDLESLPNSIVN

LRR 18 — LRR 19 — LRR 20 — LRR 21

LKLLSKLDCSGCAKLTEIPRHIGRLTSLLELSLRDSRIVNLPESIAHLSSLKSLDLSDCKKLECIPQIPPFLKQLVA

LRR 22 — LRR 23

LDCPSIRRVMPNSLVRNLSNSEEGVFKFHFTNAQQLDSGGRANIEEDARLRMTDDAYRCVFFCFPGSAVPGWFNFRG

C-JID

KGHSVTINEDLSFCSDDRLTGFALSVVFGVLDTYAMNGRYGSFSYSLTFESDDDGTQIIPNNDVLNSYFEWNGEERC

VDKDHTFVWKFNLESLRASGMSLRLCDARSFTFEISPCDYHFRLKSGITIKECGICPLYSNKKDDKYGGAVEIEESS

GSNVAQSSRDTRREDRKRKAES

Supplementary Figure 5. Sequence analysis of the protein encoded by *I* gene in BAT93-WT. Each protein domain is shaded in different colors: TIR domain (yellow), NB-ARC domain (blue), Linker domain (grey), LRR domain (green) and C-JID domain (purple). Black arrowhead indicates the TE insertion site in the corresponding DNA sequence in the BAT93-HiFi assembly.

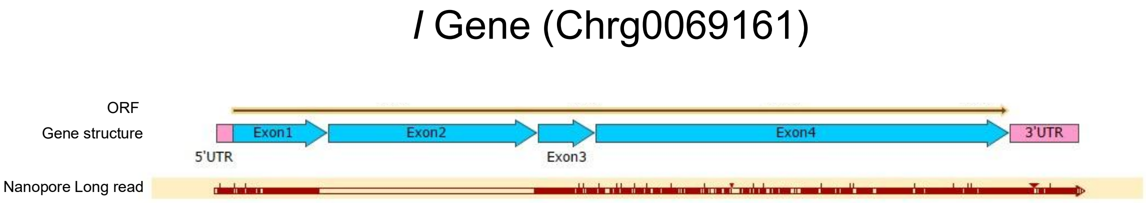

Supplementary Figure 6. Schematic representation of Nanopore long reads from BAT93-M822 transcripts mapped on the *I* gene CDS showing exon 2 skipping.

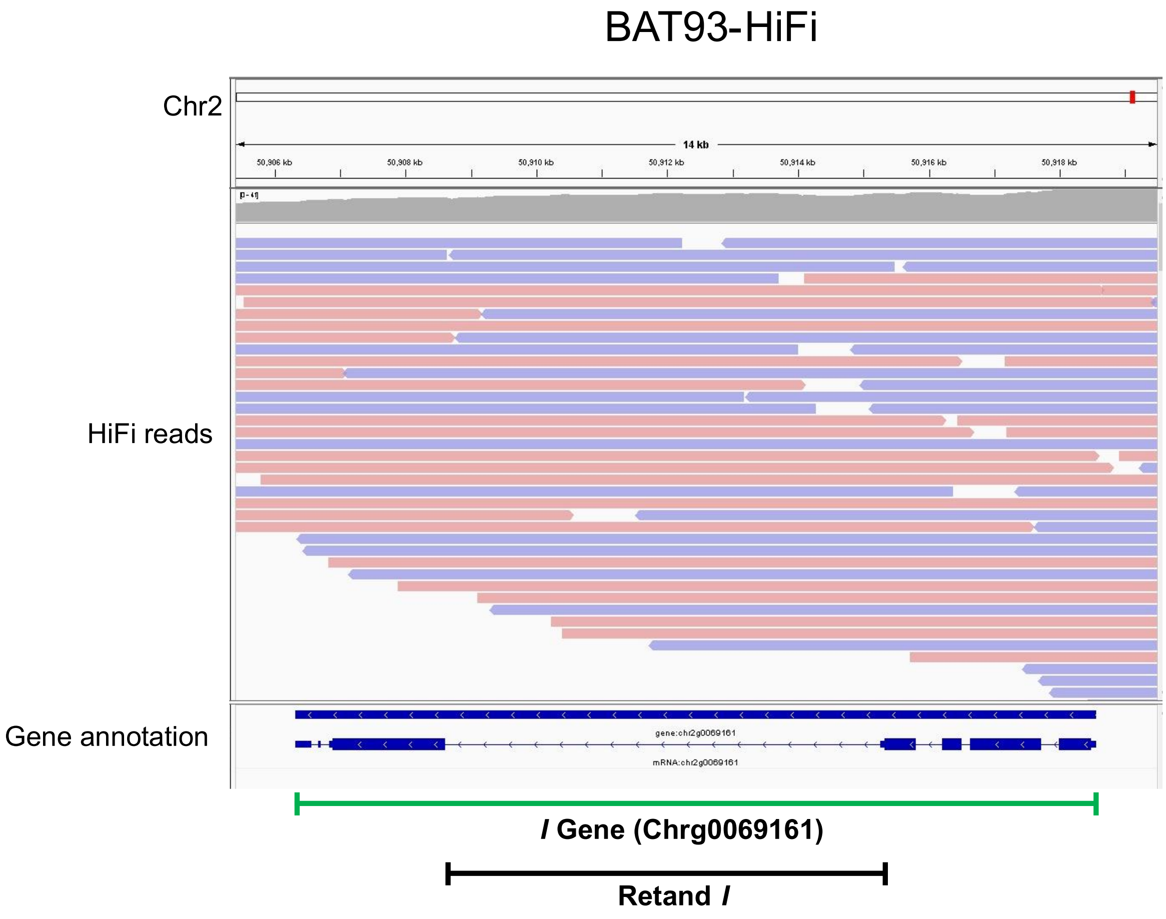

Supplementary Figure 7. Read coverage of the region containing the *I* gene in BAT93-HiFi, visualized on IGV.

a

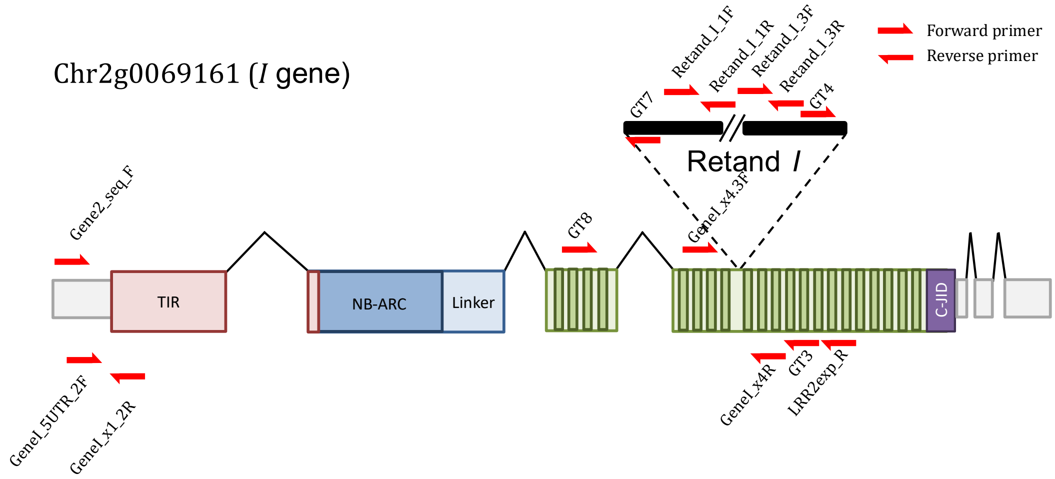

b

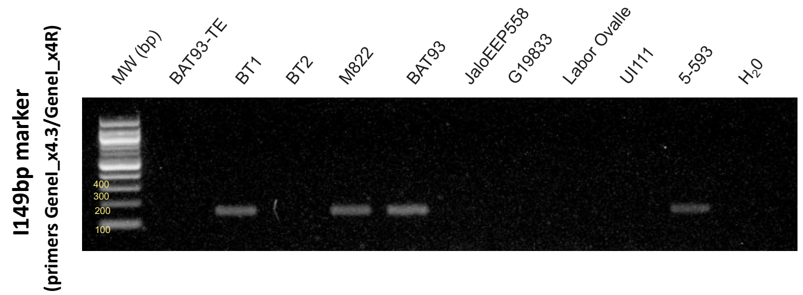

Supplementary Figure 8. Schematic representation of the *I* gene in BAT93 showing the position of the primers used in this study and the "I-149" PCR-based marker specific of the *I* gene.

a Schematic representation of the *I* gene in BAT93-TE. The position of the primers used for transcriptional analysis and genotyping of the *I* gene and Retand *I* are indicated with red arrows. **b** PCR-based marker specific of the *I* gene, referred to as "I-149" using primers GeneI_x4.3F/GeneI_x4R. Whereas amplification of a 149 bp product is detected in the BAT93 and in the EMS BAT93-M822 mutant, no amplicon is detected in the mutant BAT93-TE due to the TE insertion, testifying that this marker is specific of the *I* gene and doesn't amplify any of the remaining BAT93 31 TNL. As expected, *I* gene is also detected in 5-593 and BT1 (Black Turtle1) (corresponding to *II* genotypes), whereas it is absent in BT2 (Black Turtle 2), JaloEEP558, G19833, Labor Ovalle and UI111 (corresponding to *ii* genotypes).

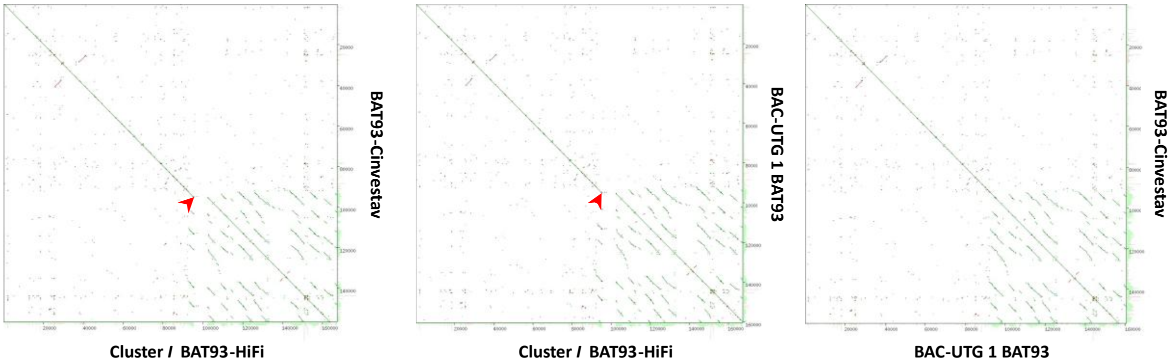

Supplementary Figure 9. Dot plot-based synteny comparison of the *I* cluster region in different sequences of BAT93.

Nucleotide sequences BAT93-HiFi, BAT93-Cinvestav assemblies and the BAC sequence UTG1 corresponding to the *I* cluster were extracted and compared by dot plot. Red arrowhead shows the TE insertion site present only in the BAT93-HiFi assembly.

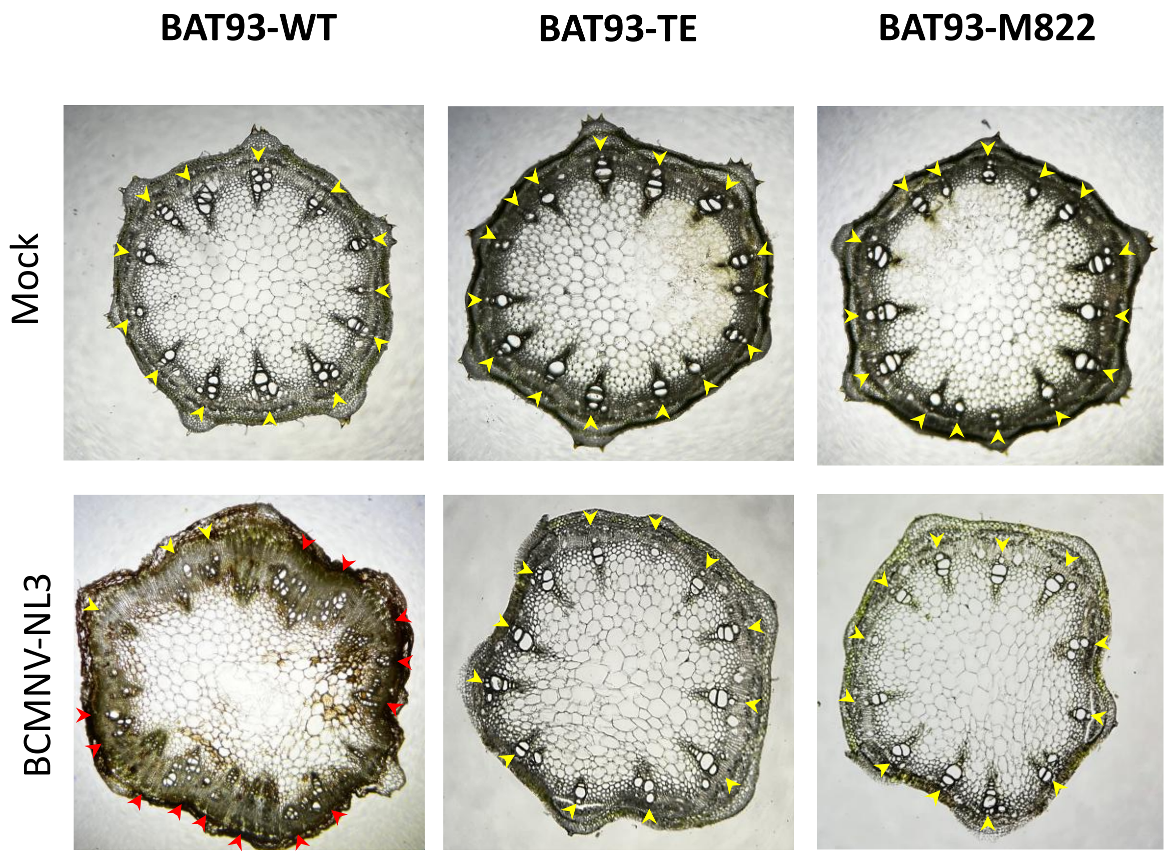

Supplementary Figure 10. Transversal stem cut from BAT93-WT and mutant plants infected with BCMNV-NL3 at 17 days post-inoculation.

Transversal cuts were performed in the stem portion below the primary leaves by using vibratome steel blades. Yellow and red arrowheads indicate healthy and necrotic phloem tissues, respectively.

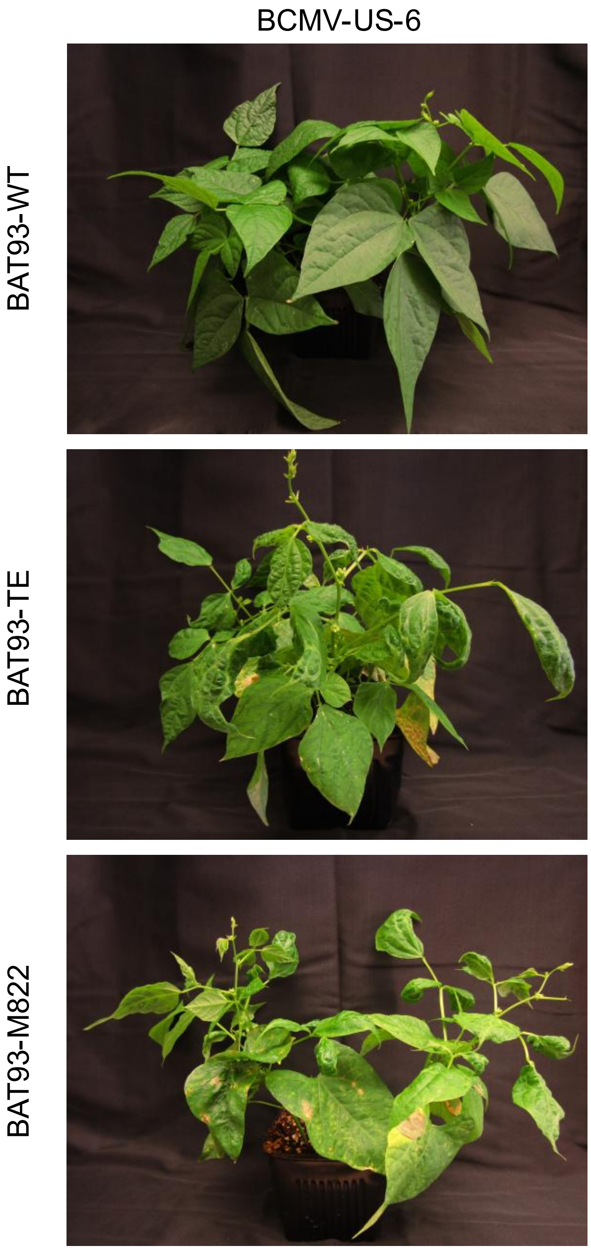

Supplementary Figure 11. Phenotypes of infection with BCMV-US-6 in BAT93-WT and mutants.

Observed phenotypes on complete plants from BAT93-WT and mutants at 21 days post-inoculation with BCMV strain US-6. Whereas no phenotype is visible on systemic leaves from BAT93-WT after BCMV infection, BAT93-TE and BAT93-M822 both present mosaic and crinkling symptoms on the trifoliate systemic leaves at 21 days post-inoculation with BCMNV strain US-6.

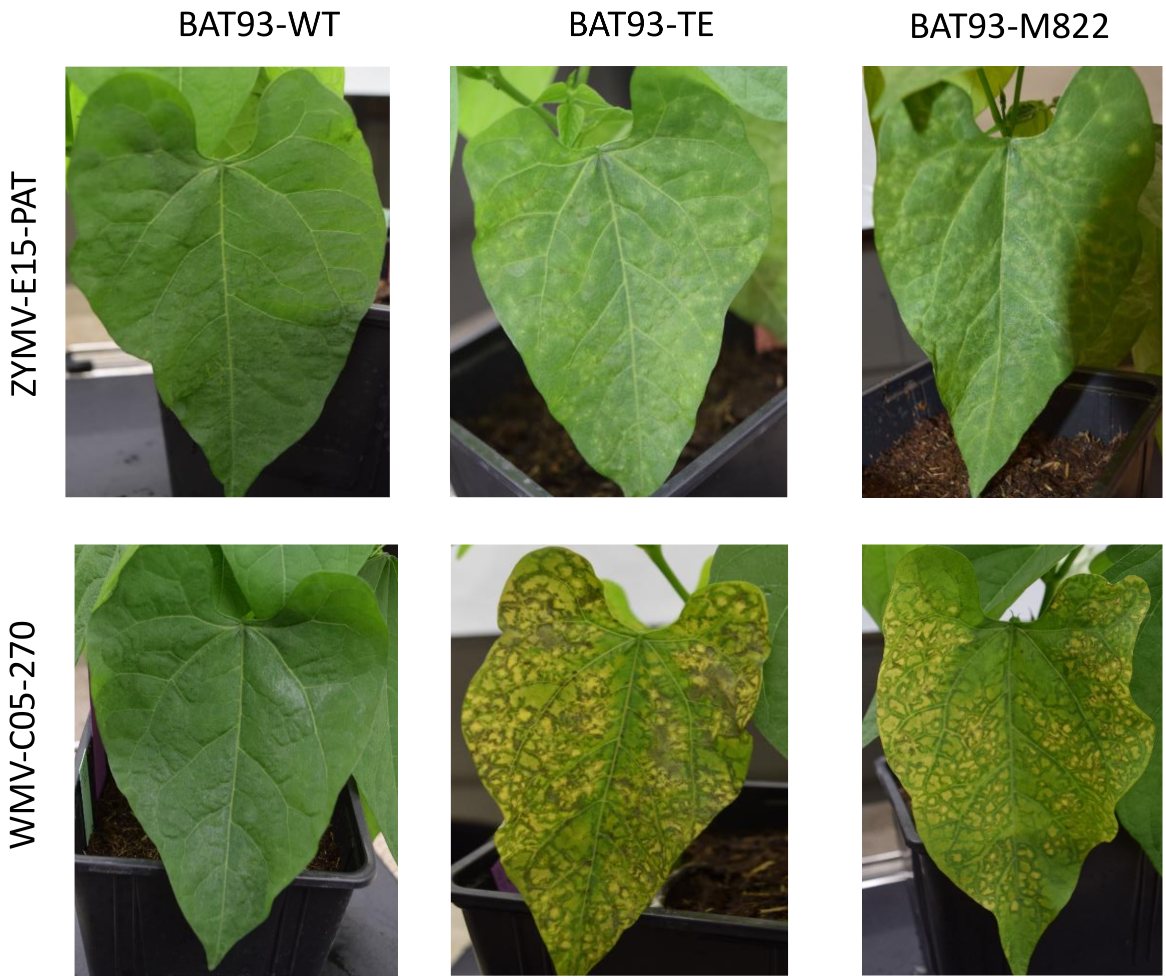

Supplementary Figure 12. Infection of BAT93 wild-type (WT) and BAT93 mutants (BAT93-TE and BAT93-M822) by ZYMV-E15-PAT and WMV-C05-270.

Primary leaves of common bean BAT93-WT, BAT93-TE and BAT93-M822, three weeks after mechanical inoculation with ZYMV-E15-PAT (top) and WMV-C05-270 (bottom). Inoculated leaves of BAT93 presented no symptoms (extreme resistance) after inoculation with ZYMV-E15-PAT or WMV-C05-270. On the contrary, both *I* mutants (BAT93-TE and BAT93-M822) presented either mosaic symptoms (ZYMV-E15-PAT) or chloronecrotic lesions (WMV-C05-270) on the inoculated leaves. The upper uninoculated leaves of all plants were all asymptomatic for ZYMV; for WMV, in the second assay, chloronecrotic areas were observed in the first uninoculated trifoliate leaves of BAT93-TE and BAT93-M822 (data not shown).

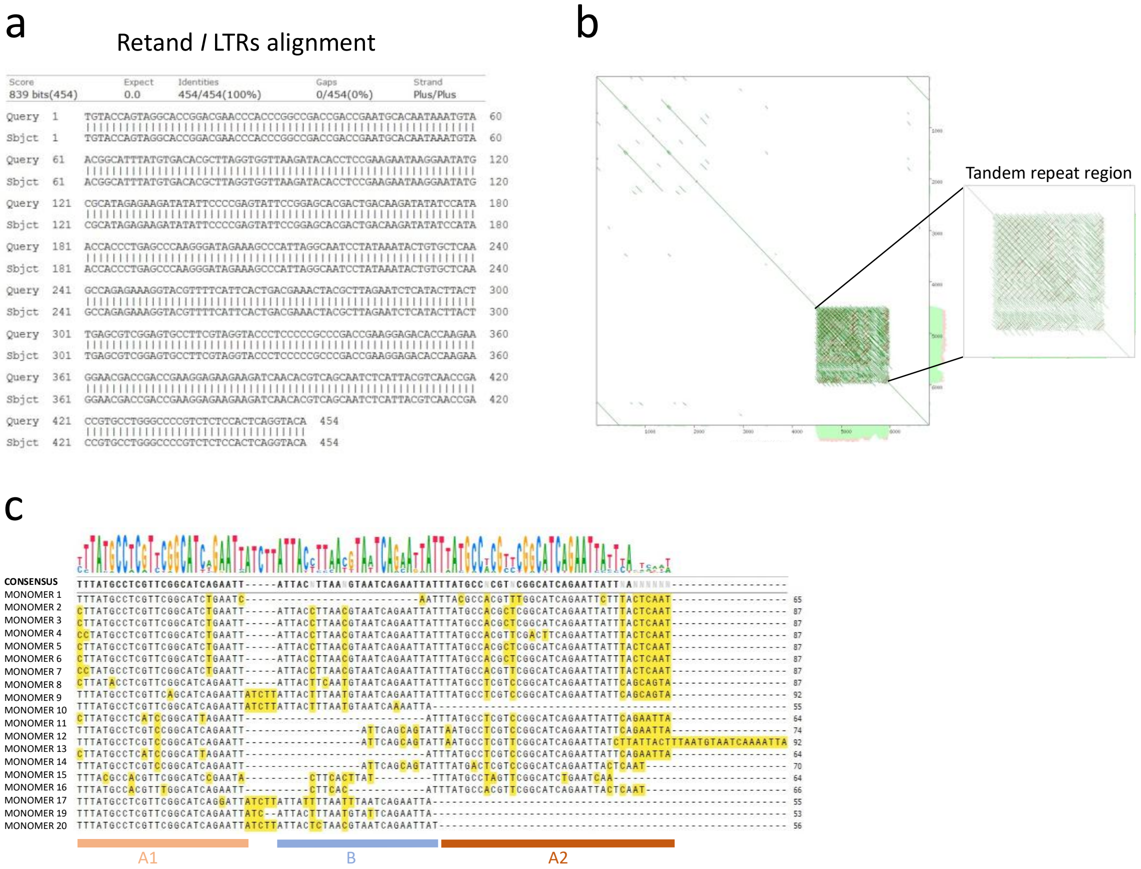

Supplementary Figure 13. Characterization of Retand *I* repetitions.

a, Sequence alignment of LTRs in the Retand *I* retrotransposon. **b**, Dot plot representation of the nucleotide sequence of Retand *I*. **c**, Multiple alignment of 20 identified monomers of the tandem repeat array. Gaps in the alignment are indicated by dashes; nucleotides that differ from prevalent bases of the consensus are highlighted in yellow and sub-repeats of tandem repeat monomers are indicated on the bottom.

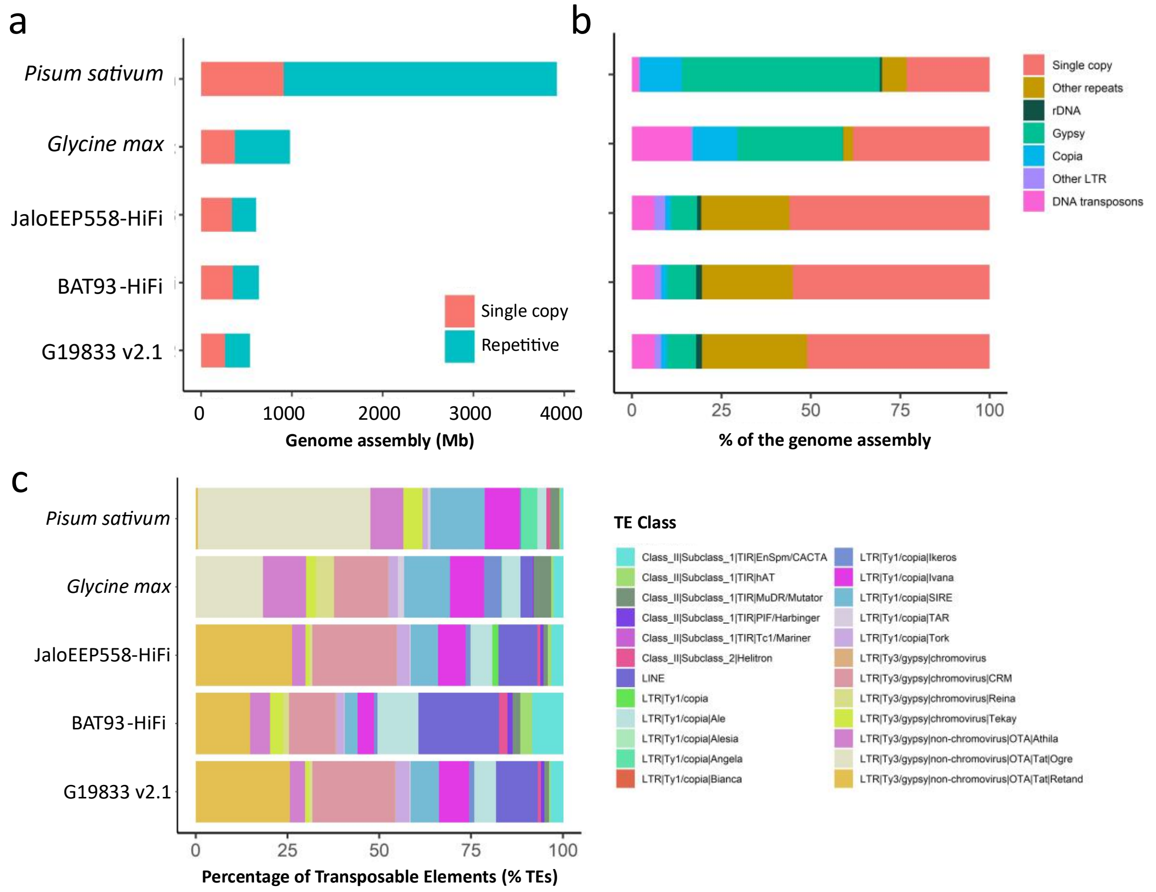

Supplementary Figure 14. Repetitive sequence composition in *P. vulgaris* genome assemblies compared with other legume genomes. a, Single copy and repeated sequence content in different genome assemblies of legume species. **b,** Proportion of single copy and repeated sequences for the different classes of repeats. **c,** Summary of the *P. vulgaris*, soybean and pea transposable elements composition by family. Source data are provided as Source Data file.

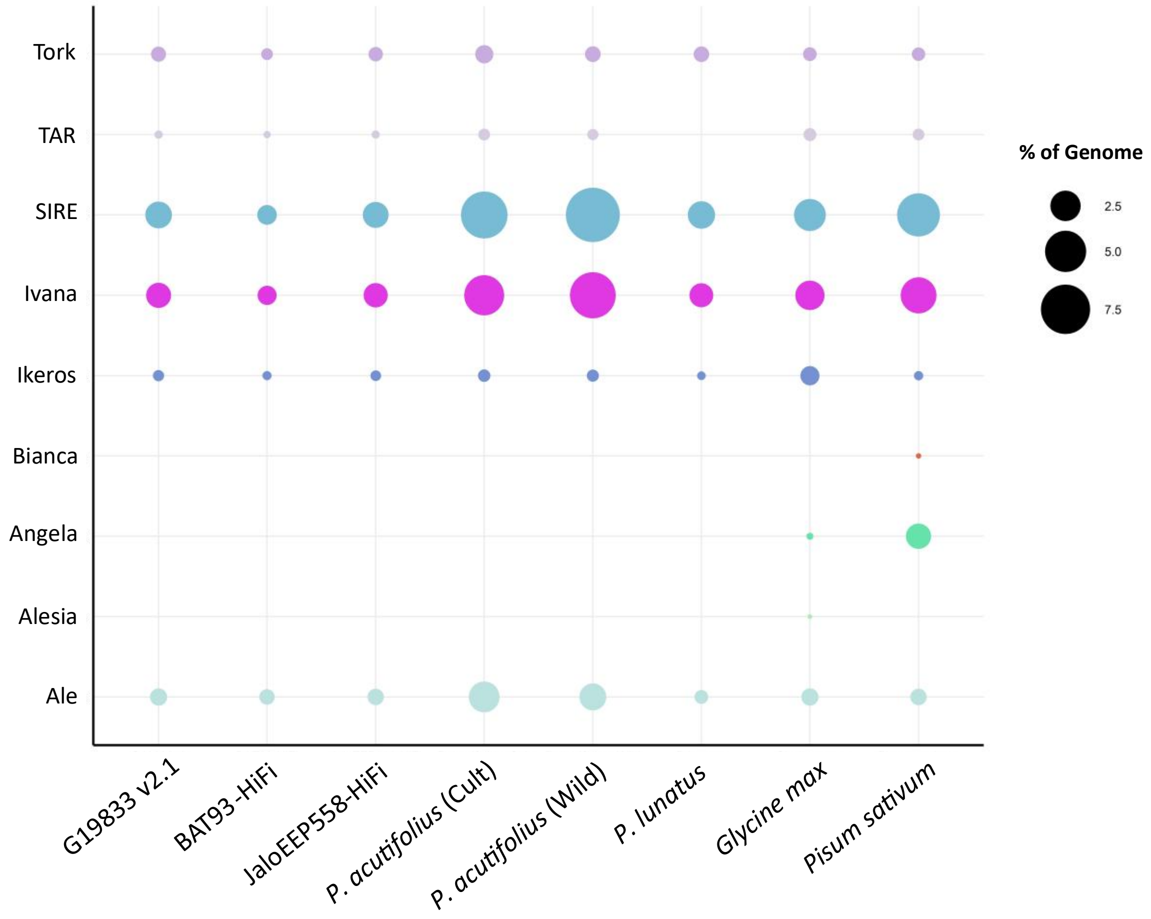

Supplementary Figure 15. Percentage of the genome occupied by the different Copia TE families in different legume genome assemblies. Source data are provided as Source Data file.

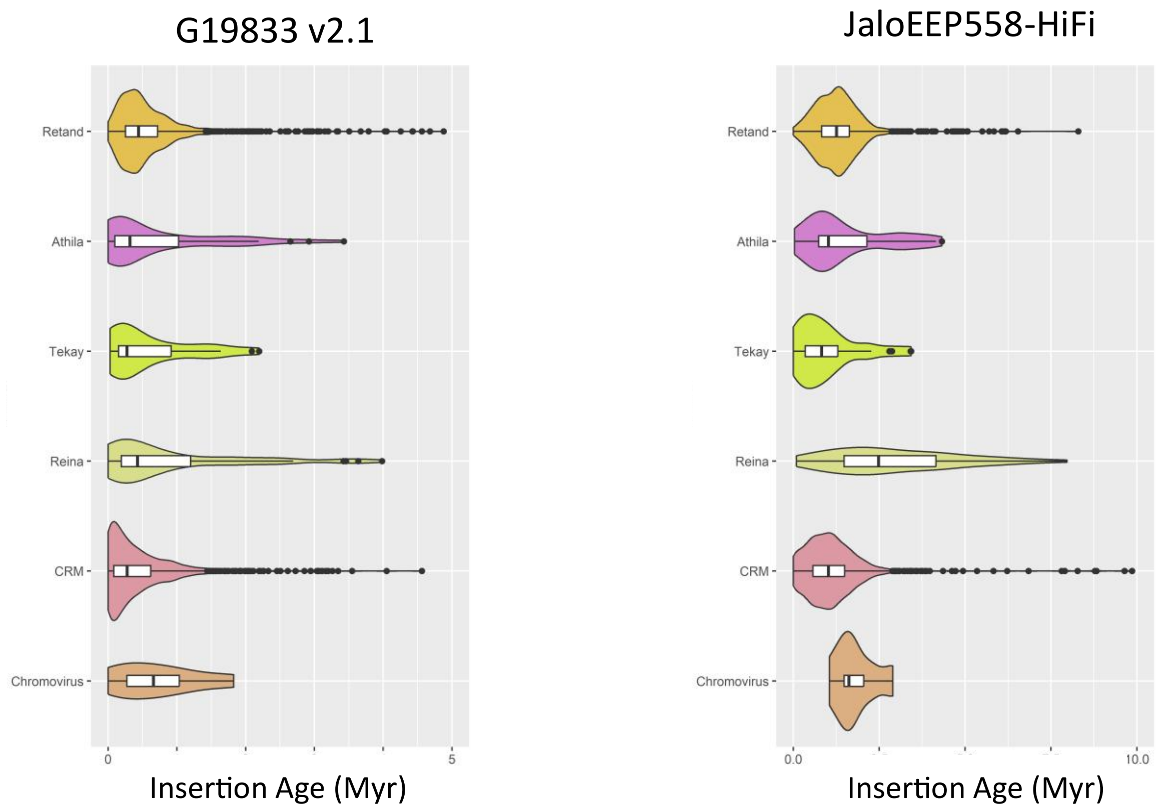

Supplementary Figure 16. Age distribution of full-length element long terminal repeat-retrotransposon (LTR-RT) Gypsy families in the G19833 v2.1 and JaloEEP558-HiFi genome assemblies. Source data are provided as Source Data file.

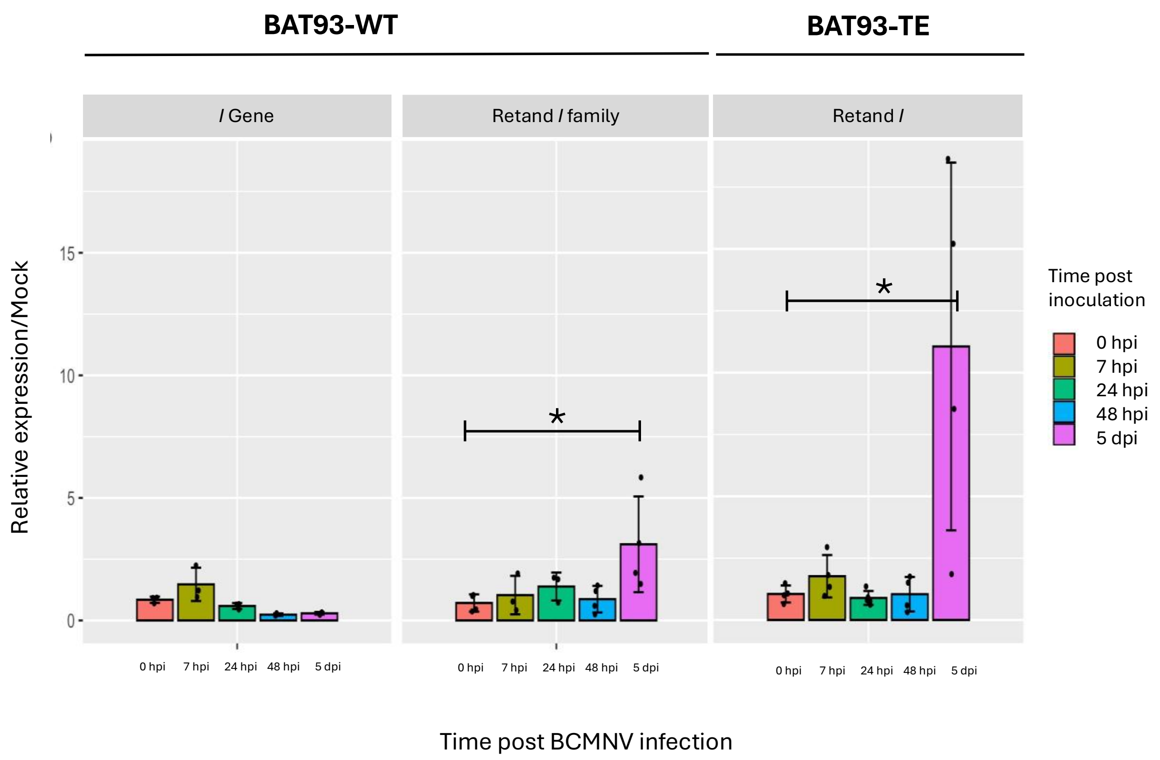

Supplementary Figure 17. Expression of the *I* gene and Retand elements in response to biotic stress (BCMNV infection).

Relative expression level of *I* gene, Retand *I* family, and the Retand *I* in BAT93-WT and BAT93-TE at different times after BCMNV infection. Data are mean ± s.d. (n = 3 independent samples, three independent experiments). Comparison between treatments was performed using the non-parametric Wilcoxon-Mann-Whitney U-test. Black dots represent individual data points. Asterisks indicate the level of significance: * $p < 0.05$, ** $p < 0.01$ and *** $p < 0.001$. Source data are provided as Source Data file.

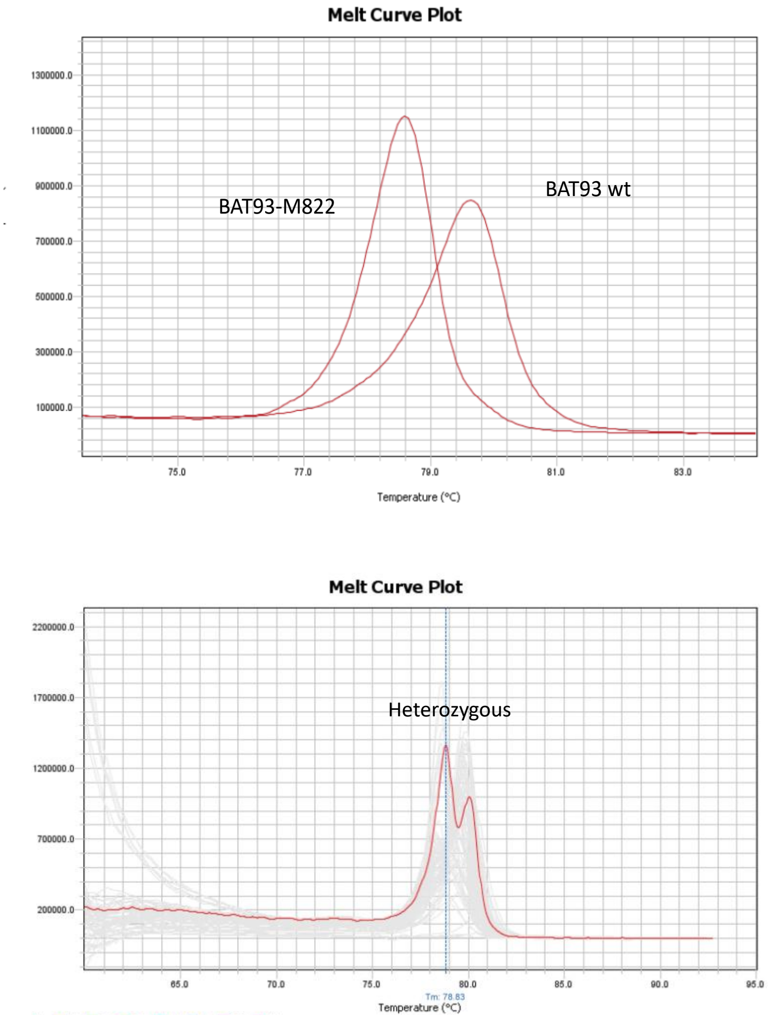

Supplementary Figure 18. Melting curve analysis of the splice acceptor variant identified in the *I* candidate gene from BAT93-M822 mutant, genotyped by the Tm-shift method.

Supplementary Table 1. Summary of transposable element composition of the genome assemblies of *P. vulgaris* based on the classification of Neumann *et al.* [1].

	BAT93-HiFi			JaloEEP558-HiFi			G19833 v2.1		
**TE Class**	**% of genome**	**Copy number**	**bp**	**% of genome**	**Copy number**	**bp**	**% of genome**	**Copy number**	**bp**
**Retrotransposon**	13,79	46128	99899900	21,85	64450	158748776	18,80	47820	143004814
LTR-Retrotransposon	12,80	41633	93938171	21,32	62584	155541504	18,29	45973	139769920
Gypsy	8,38	7825	50859793	13,93	9646	84513134	12,00	8822	76408036
Chromovirus|Unknown	0,02	46	102915	0,02	22	104951	0,02	24	104887
Chromovirus|CRM	2,28	2581	13806199	4,38	4034	26590737	3,78	3643	24080473
Chromovirus|Reina	0,09	318	527030	0,11	134	650475	0,11	141	683809
Chromovirus|Tekay	0,13	741	811908	0,14	195	855052	0,10	186	654992
Non-chromovirus|OTA|Athila	0,32	1105	1922669	0,32	637	1961731	0,32	670	2025268
Non-chromovirus|OTA|Tat|Retand	5,55	3034	33689072	8,96	4624	54350188	7,68	4158	48858607
Copia	2,59	4591	15718554	4,32	4583	26217809	4,02	4449	25613112
Ale	0,47	2283	2874850	0,53	1043	3220339	0,51	960	3262386
Ikeros	0,09	199	527611	0,15	236	894268	0,14	232	917853
Ivana	0,85	912	5177533	1,47	1324	8940352	1,37	1331	8718108
SIRE	0,93	721	5618633	1,74	1313	10553513	1,58	1255	10078489
TAR	0,03	35	209201	0,05	52	314640	0,05	52	315628
Tork	0,22	441	1310726	0,38	615	2294697	0,36	619	2320648
TRIM	0,24	6387	1510263	0,24	6219	1478292	0,28	6243	1487448
LTR-Unknown[1]	1,59	18239	10131007	2,82	37553	17114460	1,98	22010	10648212
non-LTR-Retrotransposons (LINE)	0,98	4495	5961729	0,53	1866	3207272	0,51	1847	3234894
**DNA-Transposons**	1,07	3595	6491762	0,71	1252	4311018	0,64	1134	4092085
TIR[2]	0,95	3123	5754636	0,61	1108	3715941	0,55	997	3527274
CACTA	0,68	1740	4100071	0,42	575	2553601	0,40	569	2566132
Mutator	0,13	427	793477	0,09	203	566249	0,09	205	570060
Harbinger	0,04	287	255003	0,03	151	176472	0,03	155	175729
hAT	0,10	669	606085	0,07	179	419619	0,03	68	215353
Helitron	0,12	472	737126	0,10	144	595077	0,09	137	564811
**Unknown**[3]	11,58	55590	73736087	10,74	51125	65148088	11,51	52016	61827146
**Total**	27,42	109808	186089478	33,83	118693	231415154	31,45	102817	212158939

[1] "LTR-unknown" correspond to LTR-retrotransposon unclassified between Ty1/copia and Ty3/gypsy

[2] "TIR" = Terminal Inverted Repeats

[3] "Unknown" correspond to transposable elements with conflicting annotations (e.g. DNA transposons versus LTR-RTs)

Supplementary Table 2. **HiFi sequencing statistics.**

Sequencing feature	Genotype	
	**BAT93-HiFi**	**JaloEEP558-HiFi**
Total number of CCS reads	1311207	1898624
Total size of reads (coverage)	21099762635 (31 X)	34710124064 (57X)
Mean read size (Kb)	16.1	18.3
Median read size (Kb)	15.6	17.5

Supplementary Table 3. Genomic statistics comparison in different genome assemblies of *P. vulgaris.*

Genotype	Assembly name	Sequencing/Scaffolding	Size Mb (11 Chr)	N50 Mb (L50)
G19833	Pvulgaris_441_v2.0	PacBIo RSII, Illumina/Genetic Map	537.2	49.7 (5)
BAT93	phasIbeam10.0	Roche454+Illumina+SOLiD+Sanger	494.9	0.52 (267)
BAT-Cinvestav	bat93all7-045.contigs	PacBIo RSII	566.8 (Contigs)	6.1 (29)
BAT93	BAT93-HiFi	PacBio Sequel II/Hi-C	569.4	52.3 (6)
JaloEEP558	JaloEEP558-HiFi	PacBio Sequel II/Hi-C	539.9	49.8 (6)
Labor Ovalle	PvulgarisLaborOvalle_670_v1.0	PacBio Sequel II/Hi-C	571.9	55.3 (5)
UI111	PvulgarisUI11_534_v1.0	PacBIo RSII, Illumina/ Genetic Map	554.9	51.1 (5)
5-593	5-593 v1.1	PacBio Sequel II/Hi-C	572.2	54.8 (5)

Supplementary Table 4. **Hi-C statistics.**

Sequencing feature	Genotype	
	BAT93-HiFi	JaloEEP558-HiFi
Total size of data (Gb) (Coverage)	92.5	61.1
Genome coverage	111.42 X	78.5X
Sequenced reads (paired-end)	468406039	309505651
Alignable (Normal+Chimeric Paired)	95671931	9463453
Total Hi-C Contacts	14272916	101538584
Inter-chromosomal contacts	5978856	33426554
Intra-chromosomal contacts	8294060	68112030
Short Range (<20Kb) contacts	5009406	62930506
Long Range (>20Kb) contacts	3283440	5180467

Supplementary Table 5. RNA-seq data used for annotation of BAT93-HiFi.

Sample Name	Accession/Ref	Sequencing	# Reads
Embryo + Cotyledons 48 hours old	SRR1024145	Illumina HiSeq 2000 paired end sequencing	15689781
Embryo + Cotyledons 48 hours old	SRR1025259	Illumina HiSeq 2000 paired end sequencing	10700478
Hypocotyl 48 hours old	SRR1025260	Illumina HiSeq 2000 paired end sequencing	16688099
Hypocotyl 48 hours old	SRR1025261	Illumina HiSeq 2000 paired end sequencing	12079256
Radicle 48 hours old	SRR1025262	Illumina HiSeq 2000 paired end sequencing	17240979
Radicle 48 hours old	SRR1025263	Illumina HiSeq 2000 paired end sequencing	10055529
Cotyledons 6 days old	SRR1025264	Illumina HiSeq 2000 paired end sequencing	23749232
Cotyledons 6 days old	SRR1025265	Illumina HiSeq 2000 paired end sequencing	10991311
Hypocotyl 6 days old	SRR1025266	Illumina HiSeq 2000 paired end sequencing	23773765
Hypocotyl 6 days old	SRR1025267	Illumina HiSeq 2000 paired end sequencing	11217188
Primary leaf 6 days old	SRR1025268	Illumina HiSeq 2000 paired end sequencing	18727187
Primary leaf 6 days old	SRR1025269	Illumina HiSeq 2000 paired end sequencing	8433622
Epicotyl 6 days old	SRR1025278	Illumina HiSeq 2000 paired end sequencing	19161714
Epicotyl 6 days old	SRR1025279	Illumina HiSeq 2000 paired end sequencing	8776111
Primary root 6 day old s	SRR1025280	Illumina HiSeq 2000 paired end sequencing	22147105
Primary root 6 days old	SRR1025281	Illumina HiSeq 2000 paired end sequencing	9667488
First trifoliate leaf 10 days old	SRR1025282	Illumina HiSeq 2000 paired end sequencing	18875623
First trifoliate leaf 10 days old	SRR1025283	Illumina HiSeq 2000 paired end sequencing	10525934
Hypocotyl 10 days old	SRR1025284	Illumina HiSeq 2000 paired end sequencing	17770320
Hypocotyl 10 days old	SRR1025285	Illumina HiSeq 2000 paired end sequencing	13640111
Primary leaf 10 days old	SRR1025286	Illumina HiSeq 2000 paired end sequencing	18627724
Primary leaf 10 days old	SRR1025287	Illumina HiSeq 2000 paired end sequencing	9942818
Root 10 days old	SRR1025288	Illumina HiSeq 2000 paired end sequencing	18593247
Neck of the root old 14 days	SRR1025289	Illumina HiSeq 2000 paired end sequencing	18849831
Trifoliate leaf 14 day old s	SRR1025290	Illumina HiSeq 2000 paired end sequencing	19952837
Trifoliate leaf 14 days old	SRR1025291	Illumina HiSeq 2000 paired end sequencing	5997183
Root 14 days old	SRR1025292	Illumina HiSeq 2000 paired end sequencing	9997984
Root 14 days old	SRR1025293	Illumina HiSeq 2000 paired end sequencing	18128750
Stem 14 days old	SRR1025294	Illumina HiSeq 2000 paired end sequencing	7198543
Trifoliate leaf 29 days old	SRR1025295	Illumina HiSeq 2000 paired end sequencing	9207894
Hypocotyl 29 days old	SRR1025300	Illumina HiSeq 2000 paired end sequencing	20783357
Hypocotyl 29 days old	SRR1025301	Illumina HiSeq 2000 paired end sequencing	23856591
Root 29 days old	SRR1025302	Illumina HiSeq 2000 paired end sequencing	12433138
Trifoliate leaf 35 days old	SRR1025303	Illumina HiSeq 2000 paired end sequencing	17755981
Trifoliate leaf 35 days old	SRR1025304	Illumina HiSeq 2000 paired end sequencing	21509492
Stem node 35 days old	SRR1025305	Illumina HiSeq 2000 paired end sequencing	13164438
Stem 35 days old	SRR1025306	Illumina HiSeq 2000 paired end sequencing	18720750
Stem 35 days old	SRR1025307	Illumina HiSeq 2000 paired end sequencing	22772539
Axial meristem 35 days old	SRR1025309	Illumina HiSeq 2000 paired end sequencing	9402505
Axial meristem 35 days old	SRR1025310	Illumina HiSeq 2000 paired end sequencing	20927740
Trifoliate leaf 43 days old	SRR1025313	Illumina HiSeq 2000 paired end sequencing	22866527
Trifoliate leaf 43 days old	SRR1025314	Illumina HiSeq 2000 paired end sequencing	17957896
Stem node 43 days old	SRR1025318	Illumina HiSeq 2000 paired end sequencing	30195195
Stem node 43 days old	SRR1025319	Illumina HiSeq 2000 paired end sequencing	21893505
Root 43 days old	SRR1025320	Illumina HiSeq 2000 paired end sequencing	7881321
Root 43 days old	SRR1025321	Illumina HiSeq 2000 paired end sequencing	18341351
Axial meristem 43 days old	SRR1025322	Illumina HiSeq 2000 paired end sequencing	20277370
Axial meristem 43 days old	SRR1025323	Illumina HiSeq 2000 paired end sequencing	22683255
Flower bud 50 days old	SRR1025324	Illumina HiSeq 2000 paired end sequencing	29556408
Flower 53 days old	SRR1025325	Illumina HiSeq 2000 paired end sequencing	19583364
Flower 53 days old	SRR1025326	Illumina HiSeq 2000 paired end sequencing	18472357
Small pod 60 days old	SRR1025327	Illumina HiSeq 2000 paired end sequencing	11755221
Small pod 60 days old	SRR1025328	Illumina HiSeq 2000 paired end sequencing	19011053
Medium pod 64 days old	SRR1025329	Illumina HiSeq 2000 paired end sequencing	35302797
Medium pod 64 days old	SRR1025330	Illumina HiSeq 2000 paired end sequencing	20503175
Inmature seed 79 days old	SRR1025331	Illumina HiSeq 2000 paired end sequencing	27623218
Inmature seed 79 days old	SRR1025332	Illumina HiSeq 2000 paired end sequencing	20597625
Mature pod without seeds 79 days old	SRR1025333	Illumina HiSeq 2000 paired end sequencing	13472670
Mature pod without seeds 79 days old	SRR1025334	Illumina HiSeq 2000 paired end sequencing	20040352
Mature pod 86 days old	SRR1025335	Illumina HiSeq 2000 paired end sequencing	25938804
Mature pod 86 days old	SRR1025336	Illumina HiSeq 2000 paired end sequencing	19672075
Cotyledonary leaves inoculated C531_48h_1	Alvarez-Diaz *et al.* [2]	Illumina NextSeq 500 paired end sequencing	136806899
Cotyledonary leaves inoculated C531_48h_2	Alvarez-Diaz *et al.* [2]	Illumina NextSeq 500 paired end sequencing	11930772
Cotyledonary leaves inoculated C531_48h_3	Alvarez-Diaz *et al.* [2]	Illumina NextSeq 500 paired end sequencing	12758275
Cotyledonary leaves inoculated C531_48h_4	Alvarez-Diaz *et al.* [2]	Illumina NextSeq 500 paired end sequencing	18730662
Cotyledonary leaves inoculated C531_72h_1	Alvarez-Diaz *et al.* [2]	Illumina NextSeq 500 paired end sequencing	17699543
Cotyledonary leaves inoculated C531_72h_2	Alvarez-Diaz *et al.* [2]	Illumina NextSeq 500 paired end sequencing	16643444
Cotyledonary leaves inoculated C531_72h_3	Alvarez-Diaz *et al.* [2]	Illumina NextSeq 500 paired end sequencing	11953738
Cotyledonary leaves inoculated C531_72h_4	Alvarez-Diaz *et al.* [2]	Illumina NextSeq 500 paired end sequencing	18912720
Cotyledonary leaves inoculated Cl42_48h_1	Alvarez-Diaz *et al.* [2]	Illumina NextSeq 500 paired end sequencing	12627584
Cotyledonary leaves inoculated Cl42_48h_2	Alvarez-Diaz *et al.* [2]	Illumina NextSeq 500 paired end sequencing	13418094
Cotyledonary leaves inoculated Cl42_48h_3	Alvarez-Diaz *et al.* [2]	Illumina NextSeq 500 paired end sequencing	19481756
Cotyledonary leaves inoculated Cl42_48h_4	Alvarez-Diaz *et al.* [2]	Illumina NextSeq 500 paired end sequencing	19469459
Cotyledonary leaves inoculated Cl42_72h_1	Alvarez-Diaz *et al.* [2]	Illumina NextSeq 500 paired end sequencing	105202600
Cotyledonary leaves inoculated Cl42_72h_2	Alvarez-Diaz *et al.* [2]	Illumina NextSeq 500 paired end sequencing	13154442
Cotyledonary leaves inoculated Cl42_72h_3	Alvarez-Diaz *et al.* [2]	Illumina NextSeq 500 paired end sequencing	27568827
Cotyledonary leaves inoculated Cl42_72h_4	Alvarez-Diaz *et al.* [2]	Illumina NextSeq 500 paired end sequencing	23268905

Supplementary Table 6. RNA-seq data used for annotation of JaloEPP558-HiFi.

Name	Accession	Sequencing	# Reads
PVULG_JaloEEP558-TAL18H-H2-3	SRX8819564	Illumina HiSeq 3000 paired end sequencing	26394779
PVULG_JaloEEP558-TAL18H-W2-2	SRX8819566	Illumina HiSeq 3000 paired end sequencing	18387872
PVULG_JaloEEP558-TAL18H-H2-1	SRX8819562	Illumina HiSeq 3000 paired end sequencing	29080436
PVULG_JaloEEP558-TAL18H-H2-2	SRX8819563	Illumina HiSeq 3000 paired end sequencing	30404194
PVULG_JaloEEP558-TAL18H-W2-1	SRX8819565	Illumina HiSeq 3000 paired end sequencing	15056487
PVULG_JaloEEP558-TAL18H-W2-3	SRX8819567	Illumina HiSeq 3000 paired end sequencing	16698533
Cotyledonary leaf - flg22 treated (6 hours)	This study	Illumina NextSeq 500 paired end sequencing	31966668
Root 14 days old	This study	Illumina NextSeq 500 paired end sequencing	33182259
Root 35 days old	This study	Illumina NextSeq 500 paired end sequencing	32448159
Stem 35 days old	This study	Illumina NextSeq 500 paired end sequencing	32756695
Trifoliate leaf - flg22 treated (6 hours)	This study	Illumina NextSeq 500 paired end sequencing	32360905

Supplementary Table 7. **Global annotation statistics.**

Genome annotation	Genotype	
	**BAT93-HiFi**	**JaloEEP558-HiFi**
Annotated protein-coding genes	28522	28129
non-coding RNAs	11965	12751
MIRs	124	129
tRNAs	5717	5670
rRNAs	5129	5670
Complete BUSCOs* (%)	96.7	97.0
Fragmented BUSCOs* (%)	2.1	1.9
Missing BUSCOs* (%)	1.2	1.1

*BUSCOs database: *Embryophyta* (n=1614)

Supplementary Table 8. Nanopore Long reads used to refine TNLs annotation from the *I* cluster.

Genotype	Condition (hpi*)	Organ	Sequencing	Total reads	Mapped reads
BAT93-WT	Mock	9 days old leaf	Oxford Nanopore MinION	2142871	2154394
BAT93-WT	7	9 days old leaf	Oxford Nanopore MinION	2000735	2010396
BAT93-WT	24	10 days old leaf	Oxford Nanopore MinION	790559	795030
BAT93-WT	48	11 days old leaf	Oxford Nanopore MinION	4437174	4448818
BAT93-WT	120	14 days old leaf	Oxford Nanopore MinION	2543205	2549973
BAT93-TE	0	9 days old leaf	Oxford Nanopore MinION	2322663	2327806
BAT93-M822	0	9 days old leaf	Oxford Nanopore MinION	2502264	2509016
BAT93-M822	0	9 days old leaf	Oxford Nanopore PromethION	1448538	1328144
BAT93-WT	Mock	9 days old leaf	Oxford Nanopore PromethION	862773	790000
BAT93-WT	6	9 days old leaf	Oxford Nanopore PromethION	1332795	1200597
BAT93-WT	24	10 days old leaf	Oxford Nanopore PromethION	895597	803853
BAT93-WT	NA	9 days old roots	Oxford Nanopore PromethION	1003537	972791
BAT93-WT	0	9 days old leaf	Oxford Nanopore PromethION	1062469	648125
JaloEEP558	NA	9 days old leaf	Oxford Nanopore PromethION	1360643	985546

*Hours post BCMNV-NL3 infection

Supplementary Table 9. List of primers used in this study.

Target	Primer name	Sequence (5' - 3')	Reference
I gene	GeneI_5UTR_2F	TGGTAGAAACAAAGGCACTGAGAGTT	This study
	GeneI_x1_2R	CTTTGCCCCTGACTAATCTGTCATC	
	GT8	CACCTCTTGTGGATCTGCCA	
	Gene2_seq_F	GGTAGAAATGGTAGAAACAAAGGCA	
	GT3	GAAGCATCTGCGCAAACTCA	
	LRR2exp_R	CACGGAAATCAAGCTTCCTCA	
Retand *I*	Retand_*I*_1F	AACAAGAGGTTGCATTGCACCATT	This study
	Retand_*I*_1R	TCTGTTCTGCCATAGCGTCGTTGT	
	GT7	CCCGTCTCTCCACTCAGGTA	
	GT4	GTCGTGCTCCGGAATACTCG	
Retand *I* family	Retand_*I*_3F	TAAGGCATGCCCAAATGCCAAGAG	This study
	Retand_I_3R	CCGATCATGTCGTGGAATACATGGT	
BCMV	CPF02F	ATCGGATCGAGCAAGAGA	Çelik *et al.* [3]
	CPF02R	GTCCCTTGCAGTGTGCCTTT	
BCMNV	BCMNV_3F	AAGGCCCAGCGGATAAAGACGTT	This study
	BCMNV_3R	TCGATGCACCACACCATGAAGCCA	
PvIDE	IDE qPCR F	GCAACCAACCTTTCATCAGC	Borges *et al.* [4]
	IDE qPCR R	AGAAATGCCTCAACCCTTTG	
M822v2	M822v2_Fa	gcgggcagggcggc-ATTTTCTAAATCATCGCC**A**	This study
	M822v2_R	AGGATGCAACAAGGACTGAA	
	M822v2_Fb	gcgggc-TTATTTTCTAAATCATCGCC**T**	

Supplementary references

1. Neumann, P. et al. Systematic survey of plant LTR-retrotransposons elucidates phylogenetic relationships of their polyprotein domains and provides a reference for element classification. *Mob. DNA* **10**, 1 (2019).
2. Alvarez-Diaz, J. C. et al. Genome-wide transcriptomic analysis of the effects of infection with the hemibiotrophic fungus *Colletotrichum lindemuthianum* on common bean. *Plants* **11**, 1995 (2022).
3. Çelik, A. et al. A novel study on bean common mosaic virus accumulation shows disease resistance at the initial stage of infection in *Phaseolus vulgaris*. *Front. Genet.* **14**, 1136794 (2023).
4. Borges, A. et al. Changes in spatial and temporal gene expression during incompatible interaction between common bean and anthracnose pathogen. *J. Plant Physiol.* **169**, 1216–1220 (2012).
