# Supplementary material for: The I gene defines a dynamic NLR cluster conferring broad potyvirus resistance in common bean
Source: Nat Commun. 2026 May 30;17:7042. doi: 10.1038/s41467-026-73550-x (PMC13392006; doi:10.1038/s41467-026-73550-x)
Supplement: Supplementary file 3 — Description of Additional Supplementary Files [file 41467_2026_73550_MOESM3_ESM.pdf]

Description of Additional Supplementary Files

File Name: Supplementary Data 1
Description: Gaps in BAT93 HiFi assembly

File Name: Supplementary Data 2
Description: Gaps in JaloEEP558 HiFi assembly

File Name: Supplementary Data 3
Description: Retand elements used to complete the *Phaseolus vulgaris* TE database provided by Gao *et al*. (2014).

File Name: Supplementary Data 4
Description: Assessment of centromere completeness
Identification of centromeric repeats was performed using BLASTn, followed by manual inspection of the centromeric regions with the highest density of centromeric repeats (CentPv1 and CentPv2)
