# Supplementary material for: The I gene defines a dynamic NLR cluster conferring broad potyvirus resistance in common bean
Source: Nat Commun. 2026 May 30;17:7042. doi: 10.1038/s41467-026-73550-x (PMC13392006; doi:10.1038/s41467-026-73550-x)
Supplement: Supplementary file 8 — Reporting Summary [file 41467_2026_73550_MOESM8_ESM.pdf]

nature portfolio

Corresponding author(s): Valérie Geffroy
valerie.geffroy@inrae.fr

Last updated by author(s): Aug 1, 2025

Reporting Summary

Nature Portfolio wishes to improve the reproducibility of the work that we publish. This form provides structure for consistency and transparency in reporting. For further information on Nature Portfolio policies, see our Editorial Policies and the Editorial Policy Checklist.

Statistics

For all statistical analyses, confirm that the following items are present in the figure legend, table legend, main text, or Methods section.

n/a	Confirmed	
☐	☒	The exact sample size (*n*) for each experimental group/condition, given as a discrete number and unit of measurement
☐	☒	A statement on whether measurements were taken from distinct samples or whether the same sample was measured repeatedly
☐	☒	The statistical test(s) used AND whether they are one- or two-sided<br>*Only common tests should be described solely by name; describe more complex techniques in the Methods section.*
☒	☐	A description of all covariates tested
☒	☐	A description of any assumptions or corrections, such as tests of normality and adjustment for multiple comparisons
☐	☒	A full description of the statistical parameters including central tendency (e.g. means) or other basic estimates (e.g. regression coefficient) AND variation (e.g. standard deviation) or associated estimates of uncertainty (e.g. confidence intervals)
☐	☒	For null hypothesis testing, the test statistic (e.g. *F*, *t*, *r*) with confidence intervals, effect sizes, degrees of freedom and *P* value noted<br>*Give P values as exact values whenever suitable.*
☒	☐	For Bayesian analysis, information on the choice of priors and Markov chain Monte Carlo settings
☒	☐	For hierarchical and complex designs, identification of the appropriate level for tests and full reporting of outcomes
☒	☐	Estimates of effect sizes (e.g. Cohen's *d*, Pearson's *r*), indicating how they were calculated

Our web collection on statistics for biologists contains articles on many of the points above.

Software and code

Policy information about availability of computer code

Data collection	A description of the softwares used for data collection is provided in the methods section
Data analysis	A description of the softwares used for data analysis is provided in the methods section. It includes:<br>Juicer pipeline v1.5.7<br>3D-DNA pipeline v3d-dna-529ccf4<br>Juicebox Assembly Tools v1.11.08<br>BEDTools v2.28.0<br>Circos v0.69.8<br>Mashmap (D-Genies) v2.0<br>MCScan v2<br>fastQC<br>Cutadapt<br>STAR<br>StringTie<br>EuGene release 4.2b<br>InterProScan v5.31–70.0<br>KAAS V2.0<br>KOBAS v3.0<br>BUSCOs v3.0.2

NLR Annotator v2.1
MEME v4.9.1
ARTEMIS v18.1.0
RepeatMasker v4.0.7
EDTA V1.0
R v4.1.2
SnapGene v6.2.1
MUSCLE v3.8.1
Samtools v1.9
NGSEP v4.0.2
Primer3 v0.4.0
RIdeogram (R package) v0.2.2
GraphPad Prism v8.0.1

For manuscripts utilizing custom algorithms or software that are central to the research but not yet described in published literature, software must be made available to editors and reviewers. We strongly encourage code deposition in a community repository (e.g. GitHub). See the Nature Portfolio guidelines for submitting code & software for further information.

Data

Policy information about availability of data

All manuscripts must include a data availability statement. This statement should provide the following information, where applicable:

- Accession codes, unique identifiers, or web links for publicly available datasets
- A description of any restrictions on data availability
- For clinical datasets or third party data, please ensure that the statement adheres to our policy

The raw WGS reads of BAT93-M822 generated in this study have been deposited in the National Center of Biotechnology Information (NCBI) Sequence Read Archive (SRA) under BioProject number PRJNA1042929.
The two high-quality genome assemblies for BAT93 and JaloEEP558 have been deposited in the NCBI SRA under BioProject number PRJNA1273100.
The RNA-Seq and Hi-C data have been deposited in the NCBI SRA under BioProject number PRJNA1273100.

This information is included in the manuscript.

Research involving human participants, their data, or biological material

Policy information about studies with human participants or human data. See also policy information about sex, gender (identity/presentation), and sexual orientation and race, ethnicity and racism.

Reporting on sex and gender	No human participants were involved in the research.
Reporting on race, ethnicity, or other socially relevant groupings	*Please specify the socially constructed or socially relevant categorization variable(s) used in your manuscript and explain why they were used. Please note that such variables should not be used as proxies for other socially constructed/relevant variables (for example, race or ethnicity should not be used as a proxy for socioeconomic status).*<br>*Provide clear definitions of the relevant terms used, how they were provided (by the participants/respondents, the researchers, or third parties), and the method(s) used to classify people into the different categories (e.g. self-report, census or administrative data, social media data, etc.)*<br>*Please provide details about how you controlled for confounding variables in your analyses.*
Population characteristics	*Describe the covariate-relevant population characteristics of the human research participants (e.g. age, genotypic information, past and current diagnosis and treatment categories). If you filled out the behavioural & social sciences study design questions and have nothing to add here, write "See above."*
Recruitment	*Describe how participants were recruited. Outline any potential self-selection bias or other biases that may be present and how these are likely to impact results.*
Ethics oversight	*Identify the organization(s) that approved the study protocol.*

Note that full information on the approval of the study protocol must also be provided in the manuscript.

Field-specific reporting

Please select the one below that is the best fit for your research. If you are not sure, read the appropriate sections before making your selection.

☒ Life sciences ☐ Behavioural & social sciences ☐ Ecological, evolutionary & environmental sciences

For a reference copy of the document with all sections, see nature.com/documents/nr-reporting-summary-flat.pdf

Life sciences study design

All studies must disclose on these points even when the disclosure is negative.

Sample size	This was not relevant for our study.
Data exclusions	No data were excluded from the analyses
Replication	RT-qPCR experiments were performed using at least three independent biological replicates per condition.<br>Viral infections were performed using at least two independent experiments.<br>Bioinformatic analyses were conducted using publicly available and version-controlled tools to allow reproducibility of computational workflows.<br>This is described in detail in the Methods section.
Randomization	This was not relevant for our study
Blinding	Blinding was not relevant to this study.

Reporting for specific materials, systems and methods

We require information from authors about some types of materials, experimental systems and methods used in many studies. Here, indicate whether each material, system or method listed is relevant to your study. If you are not sure if a list item applies to your research, read the appropriate section before selecting a response.

Materials & experimental systems

n/a	Involved in the study
☒	☐ Antibodies
☒	☐ Eukaryotic cell lines
☒	☐ Palaeontology and archaeology
☒	☐ Animals and other organisms
☒	☐ Clinical data
☒	☐ Dual use research of concern
☐	☒ Plants

Methods

n/a	Involved in the study
☒	☐ ChIP-seq
☒	☐ Flow cytometry
☒	☐ MRI-based neuroimaging

Plants

Seed stocks	In this study we used seed stock of cultivated common bean genotypes BAT93 and JaloEEP558 multiplicated in our greenhouse in IPS2 (France) for more than 20 years. This is described in the Methods section.
Novel plant genotypes	Two novel plant genotypes were included in this study. First, a naturally occurring mutant of the Phaseolus vulgaris genotype BAT93 was identified during the experiments and designated as BAT93-TE. Second, a mutagenized line of BAT93 referred to as BAT93-M822 was generated through EMS.
Authentication	Novel genotypes used in this study were authenticated using a combination of NGS, Sanger sequencing, and PCR with specific primers. For both BAT93-TE and BAT93-M822, sequence verification focused on confirming the presence and identity of the respective mutations. To evaluate the functional consequences of the mutations, phenotypic analyses were conducted in comparison to the wild-type BAT93. These included infections assays with the virus: BCMV, BCMNV, BPMV, ZYMV and WMV.<br>As no gene editing or transgenic approaches were used, secondary effects such as off-target edits, second-site T-DNA insertions, or mosaicism were not applicable to this study.
