# Supplementary material for: The I gene defines a dynamic NLR cluster conferring broad potyvirus resistance in common bean
Source: Nat Commun. 2026 May 30;17:7042. doi: 10.1038/s41467-026-73550-x (PMC13392006; doi:10.1038/s41467-026-73550-x)
Supplement: Supplementary file 2 — Peer Review file [file 41467_2026_73550_MOESM2_ESM.pdf]

nature portfolio

Peer Review File

I gene cloning reveals a dynamic NLR cluster conferring broad-spectrum resistance to potyviruses in common bean

Corresponding Author: Dr Valerie Geffroy

This file contains all reviewer reports in order by version, followed by all author rebuttals in order by version.

Version 0:

Reviewer comments:

Reviewer #1

(Remarks to the Author)
This study uses common bean (Phaseolus vulgaris) as a model, generating high-quality chromosome-scale genome assemblies for BAT93 and JaloEEP558, and for the first time successfully cloning and functionally validating the broad-spectrum potyvirus resistance I gene. The authors confirm that it encodes a TNL immune receptor, and reveal structural variation and evolutionary dynamics in the associated resistance gene cluster. This work provides a key genetic resource for antiviral breeding in common bean and related legumes, and offers important insights into disease resistance mechanisms. The topic is innovative, the dataset is rich, and the experimental design is generally sound; however, the following issues should be addressed to further strengthen the manuscript:
Add G19833 genome assembly metrics for comparison. Table 1 presents the assembly statistics for BAT93-HiFi and JaloEEP558-HiFi, and Figure 1c compares their synteny with the published reference genome G19833 v2.1. To facilitate direct comparison, please include the assembly statistics for G19833 v2.1 (e.g., assembly size, gap content, contig/scaffold N50) in Table 1.
Clarify gap content calculation and provide distribution. Table 1 reports gap contents of ~0.01% for BAT93-HiFi and ~0.02% for JaloEEP558-HiFi, but gap lengths are typically unknown. Please clarify the calculation method for gap proportions and provide information on the number, length distribution, and chromosomal locations of gaps to improve transparency regarding assembly completeness.
Consider achieving T2T-level assemblies. The assemblies were generated using PacBio Sequel II long reads and in situ Hi-C (lines 136–142), but the manuscript does not report the total sequencing data yield or coverage depth. Given that telomere-to-telomere (T2T) assemblies have now been achieved in several plant species, and the common bean genome is ~600 Mb, please provide the sequencing statistics and evaluate the feasibility of achieving T2T assemblies to ensure maximal reliability for downstream biological analyses.
Evaluate centromere completeness. Lines 163–164 state that G19833 v2.1, BAT93-HiFi, and JaloEEP558-HiFi show strong synteny, with only ~5 Mb of minor rearrangements near centromeric regions. However, no completeness assessment of centromeres is provided for the new assemblies. Please evaluate and report the completeness of centromeric regions and associated repeat/functional elements.
Clarify classification of NLRs as TNLs. Figure 2a shows the number of NLRs, whereas lines 180–185 discuss TNLs. Please specify how these NLRs were identified as TNLs (e.g., based on TIR domain presence, NLR annotator results) and include this classification criterion in the figure legend or methods.
Strengthen causal link for EMS mutation. The EMS mutant BAT93-M822 carries 41,310 variants compared to the wild type, with only a single base substitution identified within the 700 kb I gene cluster region. To strengthen the causal link between this mutation and the resistance phenotype, consider providing additional evidence, such as: Expanded genetic co-segregation analysis with larger populations; Functional complementation; Targeted gene editing in wild-type plants to replicate the mutation and phenotype.
Assess conservation of the I gene in other genotypes. Although chr2g0069161 was validated as the I gene, the presence of orthologous or identical resistance alleles in other resistant P. vulgaris genotypes has not been described. Comparative analysis across multiple bean accessions and related legume genomes is recommended to evaluate sequence conservation and variation, which would support its broad breeding applicability.
Explain the function of PBS and PPT. Lines 279–281 mention the primer binding site (PBS) and polypurine tract (PPT) in the context of the inserted transposable element, but their biological roles are not explained. Please briefly clarify that PBS serves as the primer annealing site during reverse transcription, and PPT functions as the initiation site for plus-strand DNA synthesis, to aid reader understanding of the TE insertion mechanism.

Reviewer #2

(Remarks to the Author)
The authors have identified the I gene for durable resistance to problematic potyviruses in common bean. There has been much interest in the identity of resistance genes at the I locus for a long time from research and applied perspectives, and this manuscript opens up a lot of new possibilities for using and understanding resistance at the I locus. The authors use genetics and advanced sequencing approaches to characterize the locus and identify independent causal mutations resulting in specific loss of resistance. The very recent insertion of a Retand retrotransposable element in I was serendipitous and led to a detailed characterization of this family of transposable elements, which was interesting for its contrasts to transposable element families in other well characterized legume systems. I enjoyed reading this manuscript and found it to be well written, except for the Supplementary Figure numbering issue detailed below. Below are a few minor comments for the authors to consider.

Line 159, should be “Supplementary Fig. 2a and d” here
Line 165, should be “Supplementary Fig. 2” here
Line 168, should be “Supplementary Fig. 2c” here
Line 193, “with the NL3”
Figure 3c, if authors can provide images of corresponding mock-inoculated plants, that would provide a better context for interpreting the systemic mosaic symptoms of the infected BAT93 mutants, although they are clearly distinct from systemic necrosis on WT.
Line 215, cite Fig. 2b here instead of Supplementary Fig. 5? Alternatively, the current Supplementary Fig. 17 does not match to any subsequent description in the text. Should Supplementary Fig. 17 actually be Supplementary Fig. 5? That would solve the Supp. Fig. numbering issue that begins at line 219.
Line 219, cite Supplementary Fig. 5 here instead of Supplementary Fig. 6? Beginning here, the numbering of Supplementary Figs. is off by one until Supplementary Fig. 18, which is correct. Authors should carefully review order and appearance of citations of Supplementary Figures throughout the text.
Since BCMNV-NL3 is presented first in the text, recommend to swap panels c and d with panels a and b in Fig. 3.
Line 264, do authors think that a second gene could confer a weak recognition resulting in the chloronecrotic lesions in response to ZYMV and WMV? This could be another nuance to add to the discussion. They only clearly say that there is another gene at this locus conferring resistance to BPMV in the discussion.
Figure 4, swap panels d and e with c, so they are cited in order that they appear in the text
Supplementary Tables are not numbered in the order they are cited

Reviewer #3

(Remarks to the Author)
In this study, the authors generated high-quality bean genomes from two genotypes BAT93 and JaloEEP558. With these genomes and the mutants, they found the point mutation or the TE insertion in the TNL gene (chr2g0069161) can change the resistance to BCMV. The results may be helpful for common bean improvement, but the MS was not well presented and organized, and some data need to be collected. See my comments below.

Major comments:
1). “with final assembly lengths of 569.4 Mb and 542.4 Mb, respectively (Table 1).”. Not sure which numbers are correct as I can see from Table 1 that the genome size is 637.8 and 606.5, respectively. For the comparison with the previous studies, it is worth noting that any new genes were annotated with the improved genomes. Please check the Fig.1b-III, I feel that the density of DNA transposons is too low? “Transposable element (TE) annotation revealed a concentration of Class I TE around the (peri)centromeric regions.” It is better to remove this, I would say “high densities of Class I retrotransposons were presented in many regions of the common bean genome.
2). The authors need to reorganize the MS and make the MS more concise and more easily readable. For example, they found a new TE insertion in the causal gene which was presented in the gene characterization section. Not understand why they have another section “Characterization of the TE inserted in the I gene” which is too long-winded and unnecessary. Similar things also happened for the Figures (Fig 2, 3) and virus-resistance evaluations.
3). Transposon annotations were poorly presented (Supplementary Table 9). How did the authors divide the annotated LTR retrotransposons into Chromovirus, CRM and other families? CRM was identified from maize and Athila was found in Arabidopsis. For the classification of transposon families, it mostly used the 80-80-80 rule. I also see “Unknown” category for both Class I and Class II TEs. What does this mean? What evidence can support if they are real TEs but not misannotations or misclassifications? The content of CACTA should be underestimated. Hard to understand for the summary of DNA TEs, what does TIR mean? Can “helitron” be grouped into TIR”. Do all the “Unknown” contain TIRs?

4). They authors need to polish the MS and correct the typos and grammar errors.

5). The authors need to check the formats of the NC journal and see if their citations and references meet the requirements.
6). Did the authors check the gene expression of chr2g0069161 between the wild type and other mutants?

Minor comments:
1). “Common bean (Phaseolus vulgaris L.) is the most important grain legume for human consumption.” Should be “one of

the most important grain legumes". Soybean, the statement may be true.
2). "These genes are often organized in clusters" should be "into clusters".
3). "these seed-transmissible viruses have spread throughout common bean production regions worldwide". I would remove "worldwide".
4). "provides protection against a broad spectrum of pathogens, including eight potyviruses" "Eight" or "Nine"?
5). "Cluster I evolution in P. vulgaris genomes". I would say "Comparison of Cluster I evolution across six P. vulgaris genomes". If the authors would like to keep the subtitle, it is better to dig deeply and provide more information about the mechanisms of CNV. I wonder if any misassembles can be found in the genomes, especially for the three incomplete genomes. Also, please check Cluster I or I Cluster in the MS, I would like to use I cluster or I gene cluster.
6). I felt a little confusion about Fig.2 and Fig.3. I would present Fig.2b as Fig.4, it will be good for readers to understand.
7). "Altogether, this led us to conclude that a TE inserted recently in BAT93 during seed propagation in our greenhouse". It is better to compare with other common bean genomes at the locus.
8). "the isolation of the I gene allowed us to design an I-based specific marker. This PCR based marker, referred to as I149bp, produced a short amplification product (149 bp)". The marker can be "I-149" or other names.
9). "Characterization of the TE inserted in the I gene". This section is poorly presented as I can see a lot of unnecessary description, such as "the TE is a class I LTR retrotransposon of the Ty3/Gypsy/Non-Chromovirus/OTA/Tat/Retand lineage". How did the authors define the lineage? I would just say "this TE is a Ty3/Gypsy LTR retrotransposon with 100% identical long terminal
repeats (LTRs) suggesting the insertion was very recent".
10). "Retand I activation". Any new insertions were detected after the stress?
11). "Plant and Virus Material" should be "Plant and Virus Materials".

Version 1:

Reviewer comments:

Reviewer #1

(Remarks to the Author)
The authors have addressed most of my previous concerns, and the revised manuscript is substantially improved. In particular, Table 1 now includes the G19833 v2.1 assembly statistics; the calculation of gap content and the sequencing depth are clarified; the basis for classifying NLRs as TNLs is now explicitly stated; and the identification of chr2g0069161 as the I gene is strengthened by co-segregation analysis together with a second independent loss-of-function allele. These revisions improve the manuscript significantly.
However, a issue still need to be resolved before acceptance. The assessment of centromere completeness remains incomplete in the manuscript itself. Figure 1b now indicates approximate centromere positions, but the more important statement in the rebuttal: that only two chromosomes per assembly have gapless centromeres. However, this is not clearly documented in the revised manuscript or in a concise supplementary summary. I recommend adding a short quantitative summary of centromeric repeat localization and centromere completeness, either in the main text or in a supplementary table/figure.
Overall, I find the study strong and the major scientific conclusions convincing. I would support publication after minor revision to address the remaining issue noted above, without the need for an additional round of external review.

Reviewer #2

(Remarks to the Author)
The authors have responded adequately to my previous comments, no additional comments.

Reviewer #3

(Remarks to the Author)
The authors have modified the MS and addressed the reviewers' comments. It can be accepted by the NC journal.

Open Access This Peer Review File is licensed under a Creative Commons Attribution 4.0 International License, which permits use, sharing, adaptation, distribution and reproduction in any medium or format, as long as you give appropriate credit to the original author(s) and the source, provide a link to the Creative Commons license, and indicate if changes were made.
In cases where reviewers are anonymous, credit should be given to 'Anonymous Referee' and the source.
The images or other third party material in this Peer Review File are included in the article's Creative Commons license, unless indicated otherwise in a credit line to the material. If material is not included in the article's Creative Commons license and your intended use is not permitted by statutory regulation or exceeds the permitted use, you will need to obtain permission directly from the copyright holder.
To view a copy of this license, visit https://creativecommons.org/licenses/by/4.0/

REVIEWER COMMENTS

Reviewer #1 (Remarks to the Author):

We thank the reviewer for the thorough and constructive evaluation of our manuscript. Below, we provide a point-by-point response to each comment and indicate the corresponding changes made in the revised version (tracked changes or highlighted in yellow).

This study uses common bean (Phaseolus vulgaris) as a model, generating high-quality chromosome-scale genome assemblies for BAT93 and JaloEEP558, and for the first time successfully cloning and functionally validating the broad-spectrum potyvirus resistance I gene. The authors confirm that it encodes a TNL immune receptor, and reveal structural variation and evolutionary dynamics in the associated resistance gene cluster. This work provides a key genetic resource for antiviral breeding in common bean and related legumes, and offers important insights into disease resistance mechanisms. The topic is innovative, the dataset is rich, and the experimental design is generally sound; however, the following issues should be addressed to further strengthen the manuscript:

Add G19833 genome assembly metrics for comparison. Table 1 presents the assembly statistics for BAT93-HiFi and JaloEEP558-HiFi, and Figure 1c compares their synteny with the published reference genome G19833 v2.1. To facilitate direct comparison, please include the assembly statistics for G19833 v2.1 (e.g., assembly size, gap content, contig/scaffold N50) in Table 1.

OUR RESPONSE: Table 1 has been completed as requested by reviewer, by adding the assembly statistics for G19833 v2.1.

Clarify gap content calculation and provide distribution. Table 1 reports gap contents of ~0.01% for BAT93-HiFi and ~0.02% for JaloEEP558-HiFi, but gap lengths are typically unknown. Please clarify the calculation method for gap proportions and provide information on the number, length distribution, and chromosomal locations of gaps to improve transparency regarding assembly completeness.

OUR RESPONSE: Gaps originate from the Hi-C based scaffolding process. 3D-DNA analysis placed and oriented contigs along the chromosomes and 1000 Ns were added between two consecutive contigs. Some contigs have been placed manually after reprocessing Hi-C links. These are separated from their neighbors by 500 Ns. The gap content (%), presented in table 1, is the number of Ns divided by the size of the assembly in base pairs.

To clarify this point, we have added the following sentences in the Methods section:

“Oriented contigs along each chromosome were separated by 1,000 Ns. Hi-C reads were realigned to scaffolds and contigs with bwa v0.7.17 (default parameters), converted to BAM with samtools sort v1.10, and indexed. The alignment file was then inspected to detect pairs of sequences with elevated interaction frequency at their ends. When visual inspection of the Hi-C contact map suggested a high probability of co-location, the contig was manually placed on the corresponding side of the scaffold, and such manually placed contigs were separated by 500 Ns (Supplementary data 2).”

In addition, we have added a supplementary data 2 presenting GAP information for both BAT93 and JaloEEP558 genome assemblies (number, chromosomal locations- beginning and end- length).

Consider achieving T2T-level assemblies. The assemblies were generated using PacBio Sequel II long reads and in situ Hi-C (lines 136–142), but the manuscript does not report the total sequencing data yield or coverage depth. Given that telomere-to-telomere (T2T) assemblies have now been achieved in several plant species, and the common bean genome is ~600 Mb, please provide the sequencing statistics and evaluate the feasibility of achieving T2T assemblies to ensure maximal reliability for downstream biological analyses.

OUR RESPONSE: The sequencing data yield and coverage depth are presented in the “DNA extraction and sequencing” section in the “Methods” section and in Supplementary Table 2. Here is the sentence: “In total, 21.09 Gb of PacBio reads with 31x physical coverage, and 34,7 Gb of PacBio reads with 57x physical coverage were generated for BAT93 and JaloEEPP558, respectively (Supplementary Table 2).”

Concerning the feasibility of achieving T2T assembly, we would like to acknowledge that both assemblies have been produced several years ago with the versions of hifiasm available at that time. The latest version of hifiasm may slightly improve assembly metrics, but it is unlikely to achieve true T2T assemblies with the current data, given the high repeat content and especially the repeat length and complexity in these genomes. In this context, the most effective strategy would be to generate ultra-long nanopore reads, which now reach error rates of around 1 %. We agree with the reviewer that, in the near future, T2T assemblies for bean are likely to become available to the community.

Evaluate centromere completeness. Lines 163–164 state that G19833 v2.1, BAT93-HiFi, and JaloEEP558-HiFi show strong synteny, with only ~5 Mb of minor rearrangements near centromeric regions. However, no completeness assessment of centromeres is provided for the new assemblies. Please evaluate and report the completeness of centromeric regions and associated repeat/functional elements.

OUR RESPONSE: We analyzed the localization and abundance of centromeric repeats by BLAST similarity searches using the previously described bean-specific repeats CentPv1 and CentPv2 (Iwata et al., 2013). This analysis allowed identification of

centromeres arrays in each chromosome. Centromeres are now indicated in Figure 1b by arrowheads, based on the distribution of CentPv1 or CentPv2 in each pseudomolecule.

Two chromosomes per genome assembly exhibit gapless centromeres, while others contain gaps, resulting in only a partial assessment of centromere completeness.

Future studies using T2T genome assemblies will enable a more comprehensive assessment of centromeric regions. While we appreciate Reviewer 1's constructive and highly relevant observation, functional analyses of centromeric regions fall beyond the scope of this study.

Clarify classification of NLRs as TNLs. Figure 2a shows the number of NLRs, whereas lines 180–185 discuss TNLs. Please specify how these NLRs were identified as TNLs (e.g., based on TIR domain presence, NLR annotator results) and include this classification criterion in the figure legend or methods.

OUR RESPONSE: We have clarified NLR classification into TNLs in the Methods by completing the sentence as follows: "Disease resistance genes on the BAT93-HiFi and JaloEEP558-HiFi assemblies were identified using NLR annotator v2.1 (Steuernagel et al. 2020) which classifies NLRs into subclasses (e.g., TNLs based on TIR domain), and using MEME (v4.9.1) (Bailey et al. 2015) with default parameters."

We have also completed in Fig. 2a legend by adding: "All the NLRs from the *I* cluster are classified as TNLs. "

Strengthen causal link for EMS mutation. The EMS mutant BAT93-M822 carries 41,310 variants compared to the wild type, with only a single base substitution identified within the 700 kb I gene cluster region. To strengthen the causal link between this mutation and the resistance phenotype, consider providing additional evidence, such as: Expanded genetic co-segregation analysis with larger populations; Functional complementation; Targeted gene editing in wild-type plants to replicate the mutation and phenotype.

OUR RESPONSE: To strengthen the causal link between the EMS mutation in chr2g0069161 and the resistance phenotype, we have tested 166 additional F2 derived from reciprocal crosses (BAT93-WT x M822 or M822 x BAT93-WT). In these 248 F2 individuals, we found perfect co-segregation between the mutation and the resistance phenotype. These data have been integrated into the revised manuscript. Furthermore, as underlined by reviewer, a single mutation was identified within the 700 kb *I* gene cluster in chr2g0069161 in mutant BAT93-M822. In addition, in the present paper, we also report a second independent allele of *chr2g0069161* harboring a transposable element insertion, which confers susceptibility. Together, this expanded co-segregation analysis and convergent evidence from two distinct loss-of-function alleles one point mutation and one TE insertion robustly establish *chr2g0069161* as the causal *I* gene, independent of background EMS variants and aligning with gold-standard criteria for mutant validation in plant genetics.

Assess conservation of the I gene in other genotypes. Although chr2g0069161 was validated as the I gene, the presence of orthologous or identical resistance alleles in other resistant P. vulgaris genotypes has not been described. Comparative analysis across multiple bean accessions and related legume genomes is recommended to evaluate sequence conservation and variation, which would support its broad breeding applicability.

OUR RESPONSE: The *I* cluster's comparative analysis with related legume genomes, such as soybean, was reported previously (Meziadi et al., 2024; TAG). Notably, soybean lacks NLRs in the syntenic region, suggesting the *I* gene's value for soybean transgenics.

Here, we compare the *I* cluster across six common bean accessions (Fig. 2a). In BAT93 and 5-593 (both *I*-bearing), the *I* gene shows 100% sequence identity, as clarified in the revised sentence below:

“Finally, comparative sequence analysis confirms *chr2g0069161* as the *I* gene, sharing 100% sequence identity with TNL *Pv5-593.02G316500* in 5-593 (*I* genotype), while no clear homolog exists in *ii* genotypes G19833, JaloEEP558, Labor Ovalle, and UI111 (Fig. 2a).”

Explain the function of PBS and PPT. Lines 279–281 mention the primer binding site (PBS) and polypurine tract (PPT) in the context of the inserted transposable element, but their biological roles are not explained. Please briefly clarify that PBS serves as the primer annealing site during reverse transcription, and PPT functions as the initiation site for plus-strand DNA synthesis, to aid reader understanding of the TE insertion mechanism.

OUR RESPONSE: We have added that PBS and PPT are “essential for retrotransposon replication”, balancing Reviewer 1's request for clarification with Reviewer 2's call for brevity in this section. The revised sentence reads:

“This TE, referred to as Retand *I*, is 6,787 bp long, and contains 100% identical long terminal repeats (LTRs) indicative of a very recent insertion event and features a primer-binding site (PBS) and a polypurine tract (PPT), essential for retrotransposon replication (Fig. 4a).“

Reviewer #2 (Remarks to the Author):

We thank the reviewer for the thorough and constructive evaluation of our manuscript. Below, we provide a point-by-point response to each comment and indicate the corresponding changes made in the revised version (tracked changes or highlighted in yellow).

The authors have identified the I gene for durable resistance to problematic potyviruses in common bean. There has been much interest in the identity of resistance genes at the I locus for a long time from research and applied perspectives, and this manuscript opens up a lot of new possibilities for using and understanding resistance at the I locus. The authors use genetics and advanced sequencing approaches to characterize the locus and identify independent causal mutations resulting in specific loss of resistance. The very recent insertion of a Retand retrotransposable element in I was serendipitous and led to a detailed characterization of this family of transposable elements, which was interesting for its contrasts to transposable element families in other well characterized legume systems. I enjoyed reading this manuscript and found it to be well written, except for the Supplementary Figure numbering issue detailed below. Below are a few minor comments for the authors to consider.

Line 159, should be “Supplementary Fig. 2a and d” here

Line 165, should be “Supplementary Fig. 2” here

Line 168, should be “Supplementary Fig. 2c” here

OUR RESPONSE: DONE. The supplementary Figures have been numbered in the order they are cited. We have carefully reviewed the order and appearance of citations of Supplementary Figures throughout the text.

Line 193, “with the NL3”

OUR RESPONSE: DONE

Figure 3c, if authors can provide images of corresponding mock-inoculated plants, that would provide a better context for interpreting the systemic mosaic symptoms of the infected BAT93 mutants, although they are clearly distinct from systemic necrosis on WT.

OUR RESPONSE:

We have completed Fig. 3 by providing images of corresponding mock-inoculated plants.

Line 215, cite Fig. 2b here instead of Supplementary Fig. 5?

OUR RESPONSE: DONE

Alternatively, the current Supplementary Fig. 17 does not match to any subsequent description in the text. Should Supplementary Fig. 17 actually be Supplementary Fig. 5? That would solve the Supp. Fig. numbering issue that begins at line 219.

OUR RESPONSE: DONE. The supplementary Figures have been numbered in the order they are cited. We have carefully reviewed the order and appearance of citations of Supplementary Figures throughout the text.

Line 219, cite Supplementary Fig. 5 here instead of Supplementary Fig. 6? Beginning here, the numbering of Supplementary Figs. is off by one until Supplementary Fig. 18, which is correct. Authors should carefully review order and appearance of citations of Supplementary Figures throughout the text.

OUR RESPONSE: DONE. The supplementary Figures have been numbered in the order they are cited. We have carefully reviewed the order and appearance of citations of Supplementary Figures throughout the text.

Since BCMNV-NL3 is presented first in the text, recommend to swap panels c and d with panels a and b in Fig. 3.

OUR RESPONSE: As correctly noted by the reviewer, we have swapped the two panels in Fig. 3.

Line 264, do authors think that a second gene could confer a weak recognition resulting in the chloronecrotic lesions in response to ZYMV and WMV? This could be another nuance to add to the discussion. They only clearly say that there is another gene at this locus conferring resistance to BPMV in the discussion.

OUR RESPONSE: We agree with the reviewer's comment and have incorporated this nuance into the manuscript. The revised sentence now reads:

"Unlike BAT93-WT, both BAT93-TE and BAT93-M822 developed chloronecrotic lesions on the inoculated leaves after infection with WMV and ZYMV. This demonstrates that the extreme resistance response of BAT93-WT to these potyviruses is due to the *I* gene, although the lack of systemic infection, particularly for ZYMV, indicates additional resistance determinants in the BAT93 genome, likely acting at distinct infection stages (Supplementary Fig. 12)."

Figure 4, swap panels d and e with c, so they are cited in order that they appear in the text

OUR RESPONSE: As correctly noted by the reviewer, we have swapped the panels d and e with c in Fig. 4.

Supplementary Tables are not numbered in the order they are cited

OUR RESPONSE: DONE. The supplementary Tables have been numbered in the order they are cited.

Reviewer #3 (Remarks to the Author):

We thank the reviewer for the thorough and constructive evaluation of our manuscript. Below, we provide a point-by-point response to each comment and indicate the corresponding changes made in the revised version (tracked changes or highlighted in yellow).

In this study, the authors generated high-quality bean genomes from two genotypes BAT93 and JaloEEP558. With these genomes and the mutants, they found the point mutation or the TE insertion in the TNL gene (chr2g0069161) can change the resistance to BCMV. The results may be helpful for common bean improvement, but the MS was not well presented and organized, and some data need to be collected. See my comments below.

Major comments:

1). “with final assembly lengths of 569.4 Mb and 542.4 Mb, respectively (Table 1).”. Not sure which numbers are correct as I can see from Table 1 that the genome size is 637.8 and 606.5, respectively. For the comparison with the previous studies, it is worth noting that any new genes were annotated with the improved genomes.

OUR RESPONSE: The Table 1 presents “Total genome assembly size (Mb)”, corresponding to all contigs (e.g. 637.8 Mb in BAT93-HiFi), and “Assembly size in Chromosomes” corresponding to contigs organized in chromosomes after HiC scaffolding (e.g. 569.4 Mb in BAT93-HiFi). Since some of the contigs are not integrated in the chromosomes, the first number is higher than the second. To clarify this point we have changed the sentence to “with chromosome assembly lengths of 569.4 Mb and 542.4 Mb, respectively (Table 1).”

Please check the Fig.1b-III, I feel that the density of DNA transposons is too low?

OUR RESPONSE: Class II TEs (DNA transposons) are present in low abundances in common bean genome (0.64-1.07% in total), so the represented density in the graph is correct. See Supplementary Table 1 for details.

“Transposable element (TE) annotation revealed a concentration of Class I TE around the (peri)centromeric regions.” It is better to remove this, I would say “high densities of Class I retrotransposons were presented in many regions of the common bean genome.

OUR RESPONSE: Class I TEs are more densely distributed in pericentromeric and proximal than in terminal chromosomal regions, an opposite pattern when compared to the distribution of genes. This is a common feature in small plant genomes that is also clear from the present data. We included arrowheads at approximate centromeric position

at each chromosome in Fig. 1b to better demonstrate this observation and change the text accordingly.

The revised sentence is: “Transposable element (TE) annotation revealed a concentration of Class I TE around the pericentromeric and proximal regions.”

The authors need to reorganize the MS and make the MS more concise and more easily readable. For example, they found a new TE insertion in the causal gene which was presented in the gene characterization section. Not understand why they have another section “Characterization of the TE inserted in the I gene” which is too long-winded and unnecessary. Similar things also happened for the Figures (Fig 2, 3) and virus-resistance evaluations.

OUR RESPONSE: The topic “Characterization of the TE inserted in the I gene” was shortened and retained the necessary information to understand the origin of this insertion:

Transposon annotations were poorly presented (Supplementary Table 9). How did the authors divide the annotated LTR retrotransposons into Chromovirus, CRM and other families? CRM was identified from maize and Athila was found in Arabidopsis. For the classification of transposon families, it mostly used the 80-80-80 rule. I also see “Unknown” category for both Class I and Class II TEs. What does this mean? What evidence can support if they are real TEs but not misannotations or misclassifications? The content of CACTA should be underestimated. Hard to understand for the summary of DNA TEs, what does TIR mean? Can “helitron” be grouped into TIR”. Do all the “Unknown” contain TIRs?

RESPONSE:

Our LTR retrotransposon classification was performed with the pipeline DANTE (Novák et al. 2024), using the REXdb reference database described in Neumann et al. (2019), which provides a systematic and phylogenetically framework for plant LTR-retrotransposon classification which goes far beyond the traditional subdivision in Ty1/copia and Ty3/gypsy superfamilies. This similarity-based classification considers phylogenetic relationships as well as distinct sequence and structural features of the elements, also giving insights into its functionality. Elements were assigned to Chromovirus, CRM, Athila, and other lineages according to this system, with the 80-80-80 rule applied only when phylogenetic resolution was not sufficient. In the “LTR-Retrotransposon” section, we have renamed the “unknown” as “LTR-unknown” to clarify that they correspond to LTR-retrotransposon unclassified between Ty1 / copia and Ty3 / gypsy.

Concerning DNA transposons, the annotation of CACTA and other DNA transposons was based on the Extensive de novo TE annotator (EDTA) pipeline (Ou et al. 2019), which is a commonly used method. They were further classified into subfamilies and lineages, using DANTE. CACTA may be underestimated using these methods, but we did not consider necessary to refine DNA transposon annotation considering the scope of this

work. We agree with reviewer that Supplementary Table 9, renamed Supplementary Table 1, was not well presented. Helitrons are DNA transposons (Class II) but lack TIRs and instead transpose via a rolling-circle mechanism (Wicker et al. 2007). Therefore, Helitrons are not part of the TIR-based superfamilies based on DANTE's classification. We have changed the presentation of Supplementary Table 9 to clarify this point. Furthermore, the "Unknown" (last line of the table) correspond to transposable elements with conflicting annotations (e.g. DNA transposons versus LTR-RTs). So, not all "Unknown" contain TIRs. Finally, "TIR" corresponds to DNA transposons with Terminal Inverted repeats.

We have re-organized the presentation of Supplementary Table 9 to clarify these points. A footnote was included in Supplementary Table 9 to explain the terms "LTR-unknown", "TIR" and "Unknown". The annotation system was also added in the title of Supplementary Table 1.

In addition, we have added the following sentence in the Methods section to give more detail of the annotation pipeline used:

"TEs were classified into subfamilies and lineages, using the Domain based Annotation of Transposable Elements program (DANTE) (Neumann et al. 2019; Novák et al. 2024). This similarity-based classification is based on phylogenetic relationships as well as distinct sequence and structural features of the elements."

4). They authors need to polish the MS and correct the typos and grammar errors.

OUR RESPONSE: We have polished the MS and corrected the typos and grammar errors.

5). The authors need to check the formats of the NC journal and see if their citations and references meet the requirements.

OUR RESPONSE: We have checked the Nature Communications format and confirmed that the citations and references meet the requirements.

6). Did the authors check the gene expression of chr2g0069161 between the wild type and other mutants?

OUR RESPONSE: Transcription of the *I* gene in the wild genotype (BAT93) and the two BAT93 *I* mutants was determined using long read (ONT). Full transcripts were recovered for the WT, and few fragmented/short transcripts were also recovered for both mutants. The presence of transcripts for these mutants is not surprising as these two mutants (a tilling mutants and a mutation cause by a TE insertion) are not deletion mutants. Nevertheless, the transcripts for M822 and BAT93-TE appear not functional as they

display exon-skipping for M822 (Supplementary Fig. 6) and only short transcripts not covering the gene for BAT93-TE (mainly restricted to the first and part of the second exon).

Minor comments:

1). “Common bean (Phaseolus vulgaris L.) is the most important grain legume for human consumption.” Should be “one of the most important grain legumes”. Soybean, the statement may be true.

OUR RESPONSE: DONE

2). “These genes are often organized in clusters” should be “into clusters”.

OUR RESPONSE: DONE

3). “these seed-transmissible viruses have spread throughout common bean production regions worldwide”. I would remove “worldwide”.

OUR RESPONSE: We prefer to retain 'worldwide' as it appropriately emphasizes the global importance of the disease.

4). “provides protection against a broad spectrum of pathogens, including eight potyviruses” “Eight” or “Nine”?

OUR RESPONSE: DONE

5). “Cluster I evolution in P. vulgaris genomes”. I would say “Comparison of Cluster I evolution across six P. vulgaris genomes”. If the authors would like to keep the subtitle, it is better to dig deeply and provide more information about the mechanisms of CNV. I wonder if any misassembles can be found in the genomes, especially for the three incomplete genomes. Also, please check Cluster I or I Cluster in the MS, I would like to use I cluster or I gene cluster.

OUR RESPONSE: DONE

6). I felt a little confusion about Fig.2 and Fig.3. I would present Fig.2b as Fig.4, it will be good for readers to understand.

OUR RESPONSE: Figure 2 presents the identification and characterization of the *I* gene and its mutant alleles (*BAT93-M822* and *BAT93-TE*), based on sequence analysis, while Figure 3 shows the phenotypes of *BAT93-M822* and *BAT93-TE* after virus infection. We prefer to keep Figure 2 as it is, since it provides a logical progression from the chromosome level to the *I* cluster and finally to the *I* gene level.

7). “Altogether, this led us to conclude that a TE inserted recently in BAT93 during seed propagation in our greenhouse”. It is better to compare with other common bean genomes at the locus.

OUR RESPONSE: As shown in Fig. 4e, a comparison of the *I* cluster across bean genotypes reveals that the Retand *I* element is inserted in the *I* gene only in BAT93-HiFi, but not in other bean genotypes such as 5-593 (which carries the *I* gene). In addition, as described in the manuscript, dot-plot analysis of gene chr2g0069161 (from BAT93-HiFi) against either BAT93 BAC clones developed by our group in 2013 (Meziadi et al., 2024) or contigs from the BAT93-Cinestav assembly showed that this TE is present only in chr2g0069161 in BAT93-HiFi (Supplementary Fig. 9). This indicates that only certain seed batches produced recently by our group at IPS2 (France) carry the Retand insertion in the *I* gene. Altogether, these findings led us to conclude that the TE inserted recently in BAT93 during seed propagation in our greenhouse.

8). “the isolation of the I gene allowed us to design an I-based specific marker. This PCR based marker, referred to as I149bp, produced a short amplification product (149 bp)”. The marker can be “I-149” or other names.

OUR RESPONSE: As suggested by the reviewer, we have changed the name of the marker to “I-149”.

9). “Characterization of the TE inserted in the I gene”. This section is poorly presented as I can see a lot of unnecessary description, such as “the TE is a class I LTR retrotransposon of the Ty3/Gypsy/Non-Chromovirus/OTA/Tat/Retand lineage”. How did the authors define the lineage? I would just say “this TE is a Ty3/Gypsy LTR retrotransposon with 100% identical long terminal repeats (LTRs) suggesting the insertion was very recent”.

OUR RESPONSE: As previously said, the topic “Characterization of the TE inserted in the I gene” was shortened and retained the necessary information to understand the origin of this insertion.

10). “Retand I activation”. Any new insertions were detected after the stress?

OUR RESPONSE: We did not check for new integrations. This will be the subject of a future publication.

11). “Plant and Virus Material” should be “Plant and Virus Materials”

OUR RESPONSE: DONE.
